# Evolutionary genetics of immunological supertypes reveals two faces of the Red Queen

**DOI:** 10.1038/s41467-017-01183-2

**Published:** 2017-11-03

**Authors:** Jackie Lighten, Alexander S. T. Papadopulos, Ryan S. Mohammed, Ben J. Ward, Ian G. Paterson, Lyndsey Baillie, Ian R. Bradbury, Andrew P. Hendry, Paul Bentzen, Cock van Oosterhout

**Affiliations:** 1 School of Environmental Sciences, University of East Anglia, Norwich, Norfolk NR4 7TJ UK; 2 Molecular Ecology and Fisheries Genetics Laboratory, Environment Centre Wales, School of Biological Sciences, Bangor University, Bangor, LL57 2UW UK; 3 Department of Life Sciences, The University of the West Indies, St Augustine, Trinidad and Tobago; 4 Earlham Institute, Norwich Research Park Innovation Centre, Colney Lane, Norwich NR4 7UZ UK; 5 Marine Gene Probe Laboratory, Department of Biology, Dalhousie University, 1355 Oxford Street, Halifax, NS Canada B3H 4R2; 6 Michael Smith Laboratories, University of British Columbia, 2185 East Mall, Vancouver, BC Canada V6T 1Z4; 7 Science Branch, Department of Fisheries and Oceans Canada, 80 East White Hills Road, St. John’s, NL Canada A1C 5X1; 8 McGill University, 859 Sherbrooke Street West, Montreal, QC Canada H3A 0C4; 9 Redpath Museum, McGill University, 859 Sherbrooke Street West, Montreal, QC Canada H3A 0C4

## Abstract

Red Queen host–parasite co-evolution can drive adaptations of immune genes by positive selection that erodes genetic variation (Red Queen arms race) or results in a balanced polymorphism (Red Queen dynamics) and long-term preservation of genetic variation (trans-species polymorphism). These two Red Queen processes are opposite extremes of the co-evolutionary spectrum. Here we show that both Red Queen processes can operate simultaneously by analysing the major histocompatibility complex (MHC) in guppies (*Poecilia reticulata* and *P. obscura*) and swamp guppies (*Micropoecilia picta*). Sub-functionalisation of MHC alleles into ‘supertypes’ explains how polymorphisms persist during rapid host–parasite co-evolution. Simulations show the maintenance of supertypes as balanced polymorphisms, consistent with Red Queen dynamics, whereas alleles within supertypes are subject to positive selection in a Red Queen arms race. Building on the divergent allele advantage hypothesis, we show that functional aspects of allelic diversity help to elucidate the evolution of polymorphic genes involved in Red Queen co-evolution.

## Abstract (web summary)

Host-parasite coevolution can lead to arms races favouring novel immunogenetic alleles or the maintenance of diversity in a balanced polymorphism. Here, Lighten et al. combine data on MHC diversity across three guppy species and simulations to show that polymorphisms of immunogenetic supertypes may persist even as alleles within supertypes are involved in an arms race.

## Introduction

Co-evolution is defined as the reciprocal, adaptive genetic changes between two or more interacting species^1^. Unlike adaptive evolution to the abiotic environment, during co-evolution the selection pressures constantly change because adaptations in one species provoke counter-adaptations in the co-evolving species. Rather than climbing a fitness peak in a nearly fixed adaptive landscape, the landscape itself is evolving in response to selection by antagonistic species climbing their respective fitness peaks. In host–parasite co-evolution, this amounts to species gaining no fitness advance despite continuous adaptions, making antagonistic co-evolution a zero-sum game. Van Valen^2^ named this dynamic co-evolutionary process after the character of the Red Queen in Lewis Carroll’s ‘Through the Looking Glass’, who said: ‘… it takes all the running you can do, to keep in the same place’. Here, we investigate the population genetic processes underpinning co-evolutionary change in the major histocompatibility complex (MHC) of three species of Poecilid fish to understand Red Queen processes of host–parasite co-evolution.

**Fig. 1:**
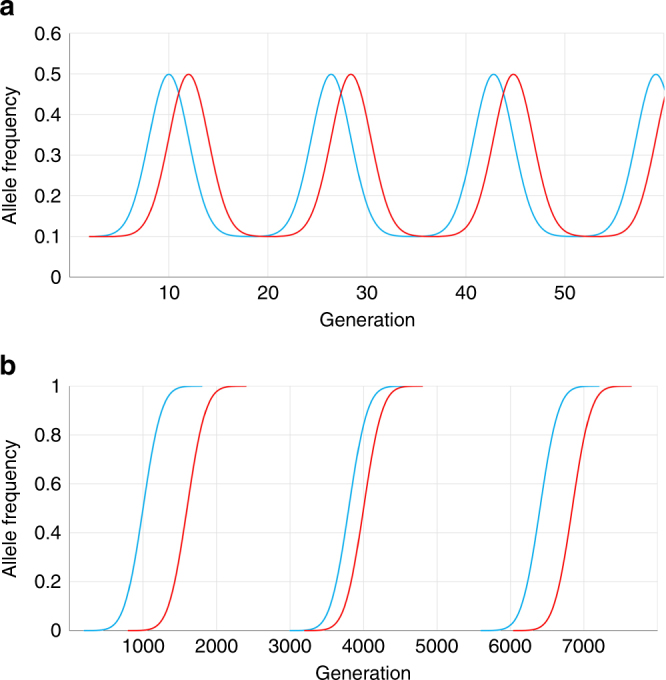
Allele frequency changes driven by Red Queen co-evolution. **a** Red Queen dynamics: a dynamic equilibrium of co-evolution acting on host immune alleles (blue) and pathogen virulence alleles (red). This is maintained by balancing selection acting on existing polymorphism over relatively short evolutionary time scales. **b** Red Queen arms race: recurrent bouts of positive selection in host and pathogen operating on new polymorphisms arising though mutation (adapted from ref. ^1^)

Co-evolution can result in stable or dynamic polymorphisms with cyclic or chaotic fluctuations in allele frequencies, i.e., Red Queen dynamics^1^ (Fig. 1a). The evolutionary force operating to produce these fluctuations is balancing selection^3, 4^. In contrast, host–parasite co-evolution can also result in the successive fixation of favourable alleles, i.e., the Red Queen arms race^1^ (Fig. 1b). Underpinning the arms race is positive selection, and unlike Red Queen dynamics, genetic polymorphisms are transient with alleles replacing each other^1^. Genetic polymorphism for some immune genes can be shared by species that have diverged by millions of years, and this phenomenon is known as trans-species polymorphism (TSP)^5, 6^. TSP is consistent with Red Queen dynamics (balancing selection)^7^, but not with a Red Queen arms race. These two Red Queen processes are opposite extremes of a co-evolutionary spectrum^8^, and it is difficult to consolidate how both processes can operate simultaneously on the same gene in a population^9^. According to Deborah Charlesworth, ‘For MHC genes, frequently observed high diversity and trans-specific polymorphism rule out a high turnover rate and, thus, arms race scenarios’^10^.

TSP has been observed in numerous immune genes^6, 11, 12^. A well-studied example of balancing selection and TSP is the vertebrate MHC, in which genetic polymorphisms (allelic lineages) can be preserved over millions of years. The most ancient case of TSP reported is for MHC class I, with allelic lineages shared between paddlefish (*Polyodon spathula*) and Chinese sturgeon (*Acipenser sinensis*), two fish species that diverged 187 million years ago^13^. The strongest signal of TSP in the MHC is in the small exonic sections that encode the peptide-binding region (PBR), which forms the adaptive interface of MHC proteins and bind the epitopes of parasite antigens^6^. Remarkably, these codons experience the most intense positive selection, evidenced by the elevated ratio of non-synonymous to synonymous substitutions (dN/dS > 1)^4^. In addition, the MHC in large meta populations is typified by allelic turnover^14–16^, which is indicative of positive selection. Like the MHC, plant resistance genes (R genes) also show both signals of selection. For example, R genes of different Arabidopsis species can evolve rapidly though positive selection^17^, yet at the same time, the signal of TSP in these genes suggests that balancing selection may help to maintain some functional variation^17, 18^.

To study Red Queen co-evolution, we genotyped MHC class IIb of 1675 fish across three species and two genera, guppies (*Poecilia reticulata* and *P. obscura*) and swamp guppies (*Micropoecilia picta*), from 59 populations in 39 rivers/streams/lake across Trinidad, Tobago, Barbados and Hawaii. We detected 539 alleles that were grouped in 15 supertypes (STs) based on the similarity in the physicochemical properties of their PBR amino acids^19^. At the macro-evolutionary scale, these STs were shared between genera diverged by >20 million years^20^, and such TSP is strong evidence of balancing selection and Red Queen dynamics^7^. However, at the micro-evolutionary scale, we observed large population genetic differentiation of the MHC alleles, which suggests adaptive evolution by positive selection and a Red Queen arms race.

**Fig. 2:**
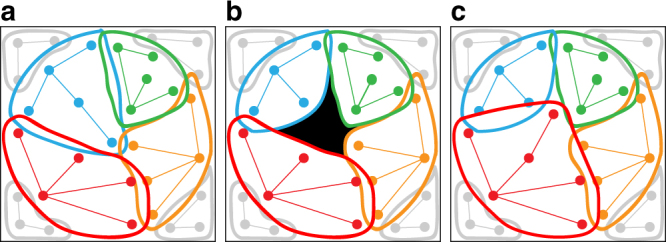
Diagrammatic representation of adaptive evolution of immune supertypes. Supertypes (STs) evolve an epitope/paratope space within a population that results in a balanced polymorphism during Red Queen co-evolution. For simplicity in depicting interactions, we visualise all alleles present in the population. Depending on the number of MHC loci, individuals possesses just a subset of these alleles and STs. Immune alleles (dots) that are closely related are presented in coloured networks (blue, green, red and orange). Alleles of the same ST cover an area in the epitope/paratope space depicted by coloured ellipses. (Non-focal STs are depicted in grey) **a** STs in a gene pool have evolved to cover the entire epitope space with little overlap. **b** The loss of one allele from the population (e.g., due to drift or positive selection on an alternative allele) opens a hole in the paratope space (black area) that becomes exploited by parasites with matching epitope. **c** Selection favours new alleles with a paratope that covers the hole, but only rarely are these substitutions made by alleles from a different ST (red allele covering the hole left by the loss of the blue allele). This causes the STs to ‘wobble’ in the epitope/paratope space. Nevertheless, changes in the paratope of STs are restricted by the presence of neighbouring STs, effectively resulting in a form of balancing selection. Hence, STs remain conserved over evolutionary time, despite the Red Queen arms race and the high turnover of their constituent alleles (see ‘Results’ section)

Here we build on the divergent allele advantage (DAA) hypothesis^21^, which proposes that diverged alleles are selectively favoured, resulting in balancing selection that can maintain genetic polymorphisms^22, 23^. We combine the DAA with contemporary ‘epitope space theory’^24, 25^, classifying MHC alleles into STs with the aim to delineate different modes of selection acting on the MHC (i.e., MHC alleles vs. MHC STs). In addition, we present a computer model to simulate the population genetics of MHC alleles and STs in an epitope/paratope space. This concept is illustrated in Fig. 2. The loss of an allele in a population by positive selection acting on an alternative allele (or genetic drift) leads to a hole in the paratope space. This hole can be exploited by parasites with matching epitope that are selected to avoid host immune recognition. Novel allelic variants that can cover this paratope hole will be introduced to the gene pool by mutation, recombination or migration, and these alleles will be positively selected as they offer immune protection against the now common parasite strain with the given epitope. Such ‘paratope holes’ appear over space and time in host populations because not all alleles (and STs) are present in an individual. In turn, the parasite’s epitope co-evolves to exploit those holes. Our simulations show fluctuations in the frequency of STs in response to parasite-mediated balancing selection, consistent with Red Queen dynamics. However, at the same time, the extant immune alleles that constitute STs are replaced by bouts of positive selection acting on novel alleles, a pattern predicted by the Red Queen arms race (Fig. 1b). Crucially, despite the transient nature of individual alleles, STs appear to be anchored in the functional epitope/paratope space, resulting in TSP. We discuss how our study may be relevant to other highly polymorphic immune/resistance genes involved in host–parasite co-evolution in both animals and plants.

## Results

### MHC alleles and supertypes

To assess the relationship among immune alleles and STs, we PCR amplified and Illumina MiSeq sequenced the PBR of MHC IIb in 1694 fish (*P. reticulata*, *P. obscura* and *M. picta*) from 59 populations. Of these, 1675 (98.87%) could be confidently genotyped using our previously published workflow^26, 27^, resulting in 539 MHC IIb alleles (Supplementary Data 1). The total number of alleles observed within an individual (*A*_*i*_) ranged from one to nine (mean (±SD) *A*_*i*_ = 3.25 ± 1.19), with mean *A*_*i*_ varying among populations from 1.00 to 4.76 (Supplementary Data 2). Among four sequencing runs, 233 replicate amplicons were sequenced across 103 individuals (2–4 independent PCR products were sequenced for each sample), and genotyping repeatability was 99.83%.

To estimate functional diversity of the MHC, observed alleles were classified into STs based on the shared physicochemical properties of the amino acids at the PBR, which are under positive selection^19^. Alleles clustered into 15 STs (Supplementary Figs. 1, 2 and Supplementary Data 1), and the number of STs within an individual (*ST*_*i*_) ranged from one to seven (mean (±SD) = 2.79 ± 0.95, Supplementary Data 2). Across all individuals *ST*_*i*_ and *A*_*i*_ were positively correlated (linear regression *P* = <0.001, *R* ^2^ = 0.73, Supplementary Fig. 3). In all but 16 individuals (<1%), each ST was represented by a maximum of two alleles, suggesting that each ST is specific to a single MHC IIb locus in guppies. Furthermore, given that there are more STs than there are MHC IIb loci in guppies, it appears that alleles from multiple STs are segregating at the same locus. To validate the robustness of estimates of functional diversity, we also inferred the number of STs in a subsample for the data set (820 individuals), comprising ~50% of randomly drawn individuals per population. In this subsample, 407 alleles were identified (76% of all alleles) that clustered into 15 STs (Supplementary Fig. 1d), which supports the robustness of well-defined functional clusters of alleles. The total number of alleles within an ST ranged from 16 (ST-1) to 55 (ST-9) (mean 35.93 ± 11.79, Supplementary Data 1 and 3). STs were, on average, found in 17.75% (±18.62) of individuals; however, ST-9 was observed in 81.64% of individuals.

Alleles of ST-9 were present in 95% of populations. Other STs were present in 17–45 populations (29–76%). The number of ST-9 alleles within populations ranged from 1 to 15 (mean 4.38). Nine unique PBR sequences (10 alleles) observed in *M. picta* were distributed among six STs shared with guppies (Supplementary Fig. 2). Across both species, there was no correlation between the number of unique ST-9 alleles and the total ST-9 frequency within populations (linear regression *P* = 0.303, *R* ^2^ = 0.01), or the number of unique ST-9 PBR amino acid sequences and the total ST-9 frequency (linear regression *P* = 0.162, *R* ^2^ = 0.03, Supplementary Fig. 4). However, the number of ST-9 alleles was significantly correlated with the number of microsatellite alleles between populations (Pearson correlation: *r* = 0.65; *P* = <0.001), indicating that ST-9 alleles are subject to genetic drift.

For all STs, the functional redundancy (*S*_r_) of their alleles was calculated. *S*_r_ is defined as the proportion of unique alleles with identical PBR amino acid sequences within an ST (Supplementary Fig. 5 and Supplementary Data 3). We hypothesised that this redundancy is subject to drift when present in the same gene pool. Across populations, however, this redundancy would contribute to genetic differentiation. ST-9 displayed the highest redundancy (*S*_r_=3.23), with 55 alleles translating to 17 unique PBR amino acid sequences, and lowest level of amino acid differentiation among PBR amino acid sequences (mean number of amino acids differences among PBRs = 3.79). Similar degrees of redundancy and cumulative ST-9 frequency were observed in *M. picta* (Supplementary Data 4). ST-6 displayed the lowest PBR redundancy (*S*_r_ = 1.22) and highest within ST differentiation (mean number of amino acids differences among PBRs = 7.75), while still comprising a relatively high number of unique alleles (44). Although ST-6 was observed in a range of populations across different geographic regions, it was notably more common in southern Trinidad, and comparatively rare in the North Slope (Fig. 2 and Supplementary Fig. 6). ST-3 was even more localised in distribution, with high frequencies (>0.20) only in North Slope populations. In the North Slope, ST-3 was represented by just 16 alleles, with an overall *S*_r_ of 1.455, which is less than half of that observed in ST-9 (3.235). Moreover, in each population, one or two unique alleles tended to dominate the cumulative frequency of this ST suggesting that allele frequencies of this ST are subject to positive selection, or drift because of functional redundancy. In turn, this could explain why ST-9 alleles are strongly correlated to microsatellite numbers. This pattern holds when analysed across all MHC alleles and all STs; genetic differentiation (*D*_est_) of microsatellites was significantly correlated with MHC alleles but not with STs (see below).

**Fig. 3:**
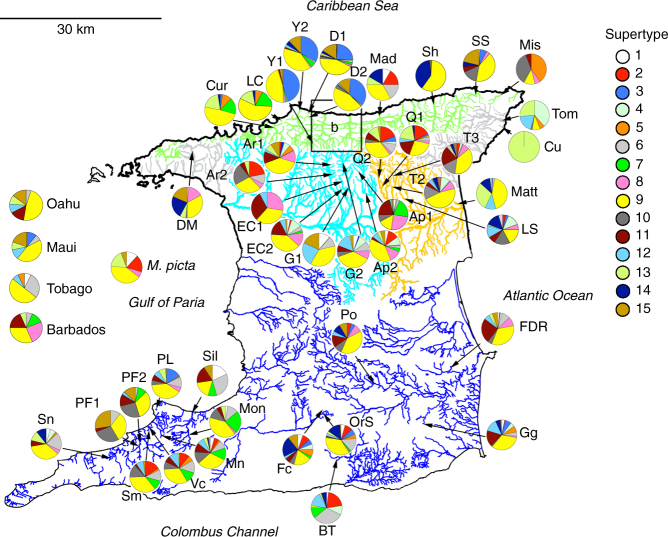
The geographic distribution of MHC supertypes (ST) in guppy populations across Trinidad and other oceanic islands. Rivers in the mountainous Northern range comprise three major regions: The North Slope (green), Caroni Drainage (light blue) and Oropouche drainage (orange). Other independent drainages in the Northern range are shown in grey. Rivers in the relatively flat regions towards the south are shown in dark blue. See Supplementary Fig. 6 for abbreviated population names and populations in region b. ST-9 is observed across 95% of populations and maintained in similar frequencies, despite wide variation in the frequencies and presence of the 55 ST-9 alleles. Importantly, *Micropoecilia picta* shares STs with the guppy, despite complete allelic divergence. ST-9 is similarly high in frequency in *M. picta*, as well as on Barbados and Hawaii, where (except for Tobago) guppies where introduced by humans. Scale – 30 km

To evaluate the evidence of TSP at the level of MHC alleles and STs, we examined two species of guppy found in Caroni drainage/North Slope (*P. reticulata*) (*n* = 790) and the Oropouche drainage/north east Trinidad (*P. obscura*) (*n* = 250)^28^. Both species share 40 (31%) alleles and 14 (93%) STs. The Caroni and Oropouche lineage share 28 (26%) alleles and 12 (80%) STs, despite at least 600,000 years of divergence with little to no gene flow^29^. (In this analysis, the introgressed Turure population^30^ was excluded). With up to three guppy generations per year, this equated to an evolutionary divergence of over ~1,800,000 generations. We further examined MHC diversity shared between both guppy species (*P. reticulata* and *P. obscura*) (*n* = 1313) and *M. picta* (*n* = 5). Despite >20 million years of divergence^20^ (~60 million guppy generations), their MHC alleles fell into STs shared with both *P. reticulata* and *P. obscura* (Fig. 3 and Supplementary Data 2). This is remarkable given that they did not have a single allele in common (Jost’s *D*_est_ = 1). Isolated, ecologically distinct guppy populations have few alleles in common while sharing a large proportion of their STs (Fig. 3 and Supplementary Fig. 6). On average, each allele was observed in only 2.08 (±SD 2.81) (3.52%) populations and 321 out of the 537 alleles (59.7%) were private (i.e., observed in only a single population). Conversely, each ST was found on average in 31.7 (±11.2) (52.1%) populations, and there were no private STs (Supplementary Data 2–4). Next, we compared MHC diversity to microsatellite diversity to quantify deviations in the MHC variation from the pattern expected under neutral evolution. Populations were highly differentiated at microsatellite alleles (*D*_est_ = 0.741 ± SD 0.007), yet the MHC alleles showed an even higher level of population differentiation (*D*_est_ = 0.88 ± SD 0.003) (Supplementary Fig. 7 and Supplementary Data 5 and 6. See Supplementary Data 8 for microsatellite genotypes). From the total of 1225 pairwise comparisons between 50 guppy populations, 1014 (82.8%) comparisons showed a higher level of differentiation for the MHC alleles than for microsatellite alleles. When correcting for non-independence and including each population only once, we found that on average only 4.3 comparisons out of 25 independent pairwise populations comparisons showed a higher *D*_est_ for microsatellite than for MHC alleles (binomial test; *P* = 0.002). The inflated level of genetic differentiation of MHC alleles is consistent with the effects of positive selection acting on these immune alleles, resulting in rapid evolutionary change and high-population differentiation. In contrast, the population differentiation based on STs (*D*_est_ = 0.388 ± SD 0.014) was significantly lower than that based on both the microsatellites, and MHC alleles (binomial tests; *P* = 7.83 × 10^−5^, and *P* = 1.75 × 10^−7^, respectively) (Supplementary Fig. 7 and Supplementary Data 5–7). This suggests that balancing selection is acting on ST variation, which homogenises ST frequencies across populations. A Mantel test with Holm *P* value correction revealed that *D*_est_ estimates of microsatellites and MHC alleles were significantly correlated (correlation = 0.10, *P* = 0.012), yet microsatellite differentiation was not significantly correlated with that of MHC STs (correlation = 0.06, *P* = 0.151). Conversely, population differentiation estimates based on MHC alleles were highly correlated with those of MHC STs (correlation = 0.43, *P* = <0.001). This further supports the hypothesis that MHC allelic variation is significantly governed by demographic processes (e.g., genetic drift), while MHC ST variation is less affected by such processes and under strong balancing selection. Simulations show that the distribution of ST variation across populations is not just an artifact of lumping alleles into groups (Supplementary Fig. 8). In other words, the simulations show that the observed ST distribution across populations is too uniform to be explained by a random process such a genetic drift, but suggests that balancing selection is homogenising the ST diversity across the guppy populations in Trinidad, Tobago, Barbados, Hawaii and *M. picta*. Such uniformity in the frequency spectrum of STs across populations is a hallmark of balancing selection^31^, and/or the presence of a consistent selection pressure (e.g., a ubiquitous parasite). Gyrodactylus species are a prevalent multicellular ecto-parasites in natural guppy populations^32^, and although infections of these parasites have been correlated to the MHC, the presence/absence of these worms was associated with a different ST^31, 33^.

Six pairwise ST combinations (out of a total of 105) show significant linkage disequilibrium (LD) after Bonferroni correction, as evidenced by the relative excess of these STs in individuals (*t*-test: *t* ≥ 3.64, *P* ≤0.001), whereas one other combination (ST-1 and ST10) shows evidence of repulsion (*t*-test: *t* = −4.59, *P* <0.001) (Supplementary Fig. 9 and Supplementary Table 1). Although various processes can cause LD (e.g., demographic fluctuations, epistatic interactions, Wahlund effects), the observation that the same STs are in LD across different populations and species suggests that these STs are physically linked on the same haploblock. Furthermore, there were significantly fewer than expected individuals with two copies of the same ST, i.e., ‘homozygous STs’ at a locus (one sample *t*-test; *t* = −10.98, *P* <0.0001), (Supplementary Fig. 10). This suggests that the polymorphism of STs is maintained by a form of balancing selection.

In summary, population genetic analysis shows that ST variation is subject to balancing selection, as is evidenced by (1) the sharing of STs among species (TSP), (2) the relatively uniform ST distribution across populations, (3) the lack of correlation with microsatellite differentiation, and (4) the deficiency of ‘homozygous STs’. In contrast, the population dynamics of MHC alleles is correlated with that of microsatellite alleles and seems to be governed at least in part by drift, as well as the effects of positive selection. Indeed, the higher level of population genetic differentiation of MHC alleles compared the (neutral) microsatellite alleles, and the absence of allele sharing (despite ST sharing) between genera is consistent with positive selection and local adaptations to parasites.

### Agent-based model of co-evolution

**Fig. 4:**
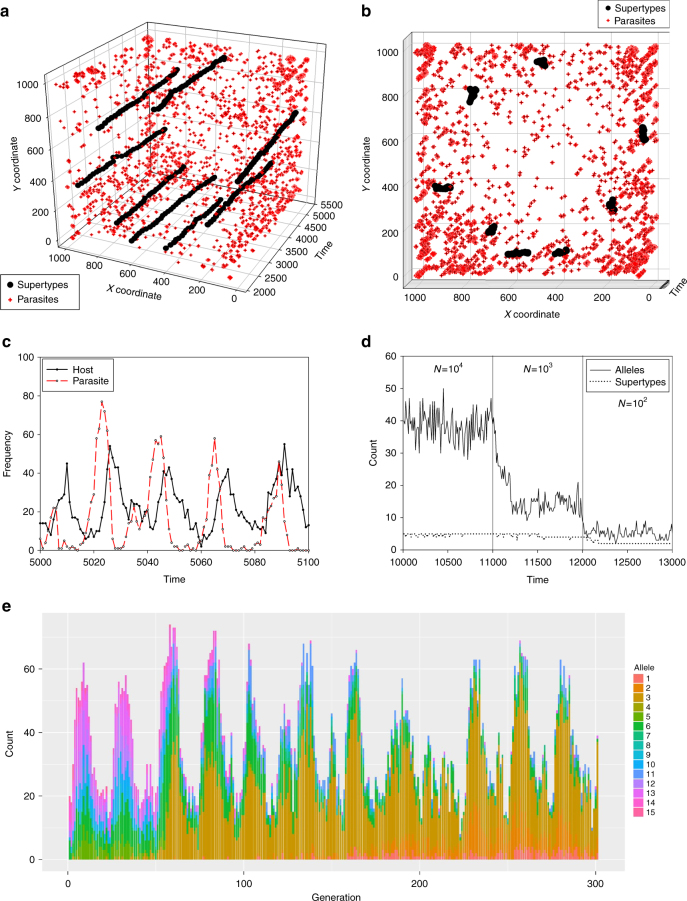
Computer simulations of host–parasite co-evolution. The centroid position of each supertype (ST) is indicated by the black dots and red crosses for parasites. Shown are simulations of a one locus model with *N* = 10,000 host population size, and mutation rate *µ* = 0.1. Eight STs evolve. **a** Each ST shows little change within the epitope/paratope space (*XY* coordinate) over time (*Z*-axis), which is consistent with trans-species polymorphism. **b** A two-dimensional view shows that STs ‘wobble’ in epitope/paratope space over time. **c** A dynamic co-evolutionary equilibrium between ST and parasite frequencies is driven by negative frequency-dependent selection, consistent with the Red Queen dynamics. **d** The number of alleles is more strongly affected by demographic processes (population sizes of *N* = 10^4^, 10^3^ and 10^2^) than the number of STs. **e** Frequencies of alleles within a single ST show a classic Red Queen arms race with alleles replacing one another over time, consistent with recurrent bouts of positive selection. STs remain preserved over evolutionary time, despite the Red Queen arms race and the high turnover of their constituent alleles (see ‘Results’ section, cf. divergent allele advantage hypothesis by Wakeland et al.^21^)

We built an agent-based model utilising ‘epitope space theory’^24, 25^ to study the evolution of immune genes in a host–parasite system (see ‘Methods’ section). In these simulations, co-evolution led to the formation and maintenance of eight equidistant STs in the finite epitope space (Fig. 4a, b). (STs were not ‘pre-programmed’ in the model; they appeared when the simulated population approached a mutation–selection–drift equilibrium). We also simulated the null model of no parasite selection (*s* = 0), which resulted in just a single ST with one (or a few) alleles that blink in and out of existence due to recurrent mutations. We observed significant fluctuations in ST frequency in response to changes in parasite frequency over time (Fig. 4c), consistent with balancing selection (Red Queen dynamics). Importantly, successive frequency peaks of a given ST can comprise different allele spectra (Fig. 4e and Supplementary Fig. 11), which is a pattern indicative of bouts of positive selection for alternative alleles (Red Queen arms race). Nevertheless, despite allelic turnover of alleles within ST (Fig 4e), the relative position of each ST remained stable in the epitope/paratope space (Fig. 4a, b). A regression analysis shows that the Euclidean distance between the centroids of STs did not change significantly over time in populations that had attained a mutation–selection–drift equilibrium (regression: F_1,23999_ = 0.52, *P* = 0.469). Indeed, the changes in allele composition and frequencies within STs merely resulted in a slight wobble in the position of STs in the epitope/paratope space when simulated over a macro-evolutionary timescale (Fig. 4a, b and Supplementary Fig. 13). Furthermore, the alleles belonging to a given ST did not stray significantly from their ST centroid position over time (Supplementary Fig. 12). Hence, despite the turbulence of the Red Queen arms race, the ST can become a TSP that coalesces deeply so that it might be shared between diverged species (Fig. 4a, b and Supplementary Fig. 12).

Finally, to assess whether demographic processes significantly affect ST diversity, we estimated the effect of strong genetic drift on both ST and allelic diversity by simulating population bottlenecks (Fig. 4e). Simulations show that although drift can greatly reduce the number of alleles, the number of STs remains comparatively constant. However, in populations with a very small effective population size (*Ne* = 100), the number of ST does go down, and each ST is represented by only one (or very small number) of alleles.

## Discussion

We genotyped major histocompatibility complex (MHC) class IIb of 1675 individuals of three species of guppies (*P. reticulata* and *P. obscura*) and swamp guppies (*M. picta*) from 59 populations in 39 rivers/streams and a lake across Trinidad, Tobago, Barbados and Hawaii. We detected 539 alleles that could be grouped in 15 supertypes (ST) based on similarities of the physicochemical properties of their peptide-binding region (PBR). The MHC IIb locus is commonly duplicated in vertebrates as a requirement to increase the immunogenetic repertoire in light of the multiple parasites that can infect an individual^4^. The birth and death^34^, and accordion model^35^ of multigene evolution, as well as empirical data^36^ suggest that copy number variation can evolve rapidly, which implies that ancient whole genome duplication that occurred in teleost fish ~350 million years ago^37^ is likely to have little impact on MHC evolution. Hence, we argue that the findings of this study are not affected by the whole genome duplication, and widely applicable for other vertebrates. Remarkably, despite being completely differentiated in terms of their alleles, the STs were shared between genera that are diverged by >20 million years^20^. Such trans-species polymorphism (TSP) is a hallmark of the MHC^11^, and the evolutionary force maintaining this diversity is balancing selection^38^. However, at the micro-evolutionary scale, we observed large genetic differentiation (expressed in Jost’s D) at the MHC alleles (but not STs), and the level of genetic differentiation even exceeded that of (neutral) microsatellites. Such a high level of genetic differentiation is evidence of spatiotemporal variation in natural selection augmenting the effects of genetic drift^15^. This interpretation is consistent with MHC studies on other species; e.g., populations of the New Zealand Hochstetter’s frog (*Leiopelma hochstetteri*) all shared the same MHC STs despite positive selection driving high population differentiation at the MHC allelic level^16^. Altogether, these data lead us to the following question: how can we explain the signal of both positive selection (rapid allelic turnover) and balancing selection (TSP of STs) at the MHC?

Brockhurst et al.^9^ defined three broad classes of Red Queen co-evolution distinguished by the modes of selection operating and the genetic architecture of co-evolving traits. Their paper discusses how both balancing and positive selection occur within the Red Queen framework, and they suggest that these modes of co-evolution often operate simultaneously in different genes. These different modes of selection result in distinctly different allele frequency dynamics (allele oscillations or recurrent bouts of positive selection)^9^. In our study, we observed a relative uniform ST distribution across populations, as well as a deficiency of ‘homozygous STs’; population genetic signatures consistent with balancing selection. On the other hand, the population genetic differentiation of MHC alleles exceeded that of microsatellites, which is consistent with spatiotemporal variation in selection and a high turnover rate of alleles due to an arms race. However, the current opinion is that the TSP of the MHC rules out a high turnover rate and arms race scenarios^10^.

Consistent with Brockhurst et al.^9^, our computer model shows that both balancing selection and positive selection can operate simultaneously, and remarkably, they can operate in synchrony on a single gene (i.e., the MHC). In our simulations, the polymorphisms of alleles cluster into groups or STs that each performs a distinct immunological function, i.e., a particular area in the epitope/paratope grid. We hypothesise that the balanced polymorphism at each MHC locus is generated by selection on alleles of different STs that are not functionally equivalent. Because an allele of a given ST cannot perform the function of an allele belonging to another ST, the two alleles cannot substitute each other at a locus without causing some dysfunction that leads to a fitness cost. In other words, the replacement of alleles in recurrent bouts of positive selection takes place within STs, whereas different STs are maintained by balancing selection driven by the necessity to broadly cover the epitope space (when viewed at the level of the population, not the level of an individual). The simulations show that despite the rapid evolution of alleles, STs show very little net evolutionary change (Fig. 4). This can be understood when realising that a significant shift of a ST in one direction (e.g., due to the loss of one of its alleles) would expose a hole in the paratope between the STs at the population level (Fig. 2). Such holes can be exploited by parasites, allowing them to infect hosts more efficiently, thus resulting in a reversal of this change, e.g., by a mutation, migration or recombination that replaces the lost allele. Each ST thus ‘wobbles’ in a given position in the epitope/paratope space, which can explain the phenomenon of TSP, and the ‘trench warfare’ hypothesis in plant resistance genes^18^. Importantly, although we simulated a fixed epitope space for simplicity, alterations in parasite community structure will result in dynamic change in the shape and size of the epitope space. Rather than climbing a fitness peak in a nearly fixed epitope space, the space itself is evolving rapidly in response to selection by antagonistic parasites attempting to climb their respective fitness peaks.

Note that there is space between the alleles of different STs, which is not presented in the simplified schematic of Fig. 2. However, in our computer simulation model, alleles are able to bind parasites even if their epitope is not exactly matching the parasite’s paratope—and this in line with empirical data^39^ and the DAA hypothesis^21^. In our model, the distance between the allele and parasite in the epitope/paratope space is used to calculate the probability of binding. Hence, even with the holes between the STs, parasites are bound by the alleles. If the distance between STs would be larger, there would be more space for the parasites to exploit, i.e., areas in the epitope/paratope grid where they would be bound less efficiently. In shorthand, we have referred to this as ‘parasites exploiting the hole in the epitope space’. We propose that this could explain the observation of TSP of STs.

Our empirical data and simulations are consistent with the DAA hypothesis of immunogenetic evolution^21^, and our computer simulations build on this. Wakeland et al.^21^ hypothesised that the preservation of diverse allelic lineages reflects the selective advantage of maintaining a broad spectrum of MHC functionality (heterozygote advantage), which has been supported by simulations of binding prediction^40^, and in the genetics of natural populations^41^. Moreover, they hypothesised that balancing selection not only operates on the presence/absence of STs (‘immune void overdominance’), but also separately on the alleles within each STs (which in their terminology was referred to as an ‘allelic lineage’). Our simulations refine the DAA model, showing that positive selection (and genetic drift) operates on alleles independent of balancing selection operating on STs. As such, we show that both Red Queen processes can operate simultaneously, even in a single locus. Furthermore, Wakeland et al.^21^ observed that although the majority of differences among alleles within each lineage were attributed to point mutations, recombination among variants also contributed to variation among alleles^21, 42^. Note, however, that we did not simulate recombination in our computer model (because that approach enabled us to define STs and allocate alleles based on their co-ancestry). However, sequence exchange through recombination between alleles of different STs could dramatically shift the paratope of the recombinant alleles, more so than any single mutation in our model. Consequently, the MHC in natural systems may be less preserved than in our simulations. Given that recombination (including gene conversion and micro-recombination) is thought to play an important role in MHC of some species^36, 42^, it would be interesting to examine the effects of recombination on the effects of TSP in future simulation studies of MHC STs.

Our study assumes that the alleles of multiple STs segregate at a single MHC locus. Some species, however, may possess loci with alleles belonging to just a single ST. Such species cannot maintain a balanced polymorphism, but they can nevertheless be polymorphic for their MHC if they possess multiple duplicated MHC loci. This genetic architecture is likely to benefit species that undergo severe inbreeding. For example, the self-fertilising fish, *Kryptolebias marmoratus* was found to have 3.9 MHC STs per individual after more than 10 generations of selfing in the laboratory, which was similar to the number found in the natural population^43^. Although MHC gene duplication can preserve the MHC polymorphism in the face of severe drift and inbreeding, this genomic architecture may also incur a possible fitness cost, such as delimiting T-cell diversity and reducing the efficiency of pathogen recognition^44^.

The existence of MHC STs has been recognised by immunologists since the mid-1990s^45^, and our study expands the evolutionary genetic implications of such sub-functionalisation. The human MHC class I, or human leukocyte antigen (HLA) alleles are traditionally clustered and defined into nine different STs, and although different methodologies have been employed to classify STs, e.g., refs. ^39, 46^, the classification of alleles into STs is broadly consistent across these methods. Each ST is characterised by a supermotif that reflects the broad main anchor motif. The majority of HLA STs demarcate groups of alleles with non-overlapping repertoires, although the binding repertoire does overlap in a small proportion of the alleles that span multiple STs^39^. Similarly, up to 62% of foreign peptides have been shown to be bound by more than one ST^39^. Nevertheless, despite some fuzziness in peptide binding and allele classification, the HLA ST classification has been effectively used to identify T-cell epitopes from many disease targets, and STs show specific disease associations, explaining variation in susceptibility and disease outcome (reviewed in ref. ^39^). The population genetic patterns of STs infer that broad MHC/HLA functionality has been driven by pathogen-mediated selection not only in humans^47^, but also in populations of non-model vertebrates^31, 48, 49^. Interestingly, when we employed a computer simulation model to study the evolutionary genetics of the MHC, alleles also clustered into groups with unique immunological function. These clusters resemble STs, and crucially, these were not ‘pre-programmed’ but they appeared because of the effects of selection, mutation and drift simulated in the agent-based model. In other words, the similarities in peptide-binding specificities of the simulated alleles within STs were the result of common ancestry and balancing selection, as has been found in the MHC^50^.

A puzzling observation about MHC gene evolution is that some studies report that MHC diversity is primarily affected by drift^51–53^, whereas others show that the high polymorphism is maintained by balancing selection^54, 55^. This contradiction is reconciled when realising that genetic drift (as well as selection) acts on the alleles, whereas balancing selection acts on the immunological function of the alleles, defined by their ST. Indeed, a previous study of Galápagos mockingbirds inferred a significant effect of genetic drift on the number of alleles in island populations but not the number of STs^56^. Our simulations confirm that the effects of drift are most noticeable after bottlenecks in populations with high allelic variation, when each ST is represented by multiple (functionally more-or-less equivalent) alleles, like the guppy’s ST-9 alleles. Our simulations demonstrate that drift during a bottleneck can erode such allelic diversity within STs with less effect on the number of STs. This is also supported by our empirical data, which shows that although there are large differences in the number of MHC alleles between populations (coefficient of variation, CV = 0.60), the number of STs is more similar (CV = 0.38). In addition, the diversity of MHC alleles (but not STs) in populations is correlated with microsatellite diversity, which shows how drift is a significant force at this level of polymorphism, a finding echoed by other MHC studies (e.g., ref. ^51^). These observations support an important prediction of Wakeland et al.^21^, in that DAA will augment rare-allele advantage and protect rare-allelic lineages (or STs) from extinction. Although the total number of alleles may be rapidly reduced in an ST by genetic drift, as it becomes increasingly rare, this ST is likely to be saved from extinction by the functional benefits it provides in recognising pathogens that are adapted to avoid recognition by other, more common STs.

Finally, our model can also explain why natural selection is unable to remove the large number of disease-causing mutations in the human MHC (HLA) that result in over 100 heritable disorders^57^. Our empirical data show there is a significant deficiency of individuals with two allelic copies of the same ST. As a consequence of this homozygote ST deficiency, recessive deleterious mutations are rarely exposed to purifying selection, potentially resulting in the buildup of a ‘sheltered load’ in a Muller’s Ratchet type process^58^ in each ST lineage. We hypothesise that each ST may thus accumulate a unique ‘sheltered load’ of recessive deleterious mutations over time^58^, which could explain the large number of heritable disorders associated to the human MHC^57^.

The implications of this study are likely to be relevant also to other genes that show high levels of allelic polymorphism despite being involved in a Red Queen arms race. Prime examples of such genes are killer cell immunoglobulin-like receptor (KIR) genes, which show extensive polymorphism^59^, plant resistance genes (R genes) that are engaged in ‘trench warfare’ and stuck in an evolutionary stalemate^18^, self-incompatibility *S*-loci in flowering plants that display patterns of diversity consistent with Red Queen dynamics and TSP^60^, and possibly also some avirulence and effector genes (see ref. ^12^ for more examples). The identification of sub-functionalised groups of alleles into STs is likely to help evolutionary genetic studies of such genes, and we believe that by studying the population genetics of STs and alleles, new light can be shed on complex observations associated with Red Queen co-evolution in immune genes.

## Methods

### Sampling

Guppies (*Poecilia reticulata*, n genotyped = 1425 and *Poecilia obscura*, n genotyped = 250) were collected between 2008 and 2012 from 59 populations distributed among 39 rivers/streams and a lake, across Trinidad, Tobago, Barbados and Hawaii. Only guppies collected in the Oropouche drainage and north east Trinidad were considered *P. obscura* ^28^. Each fish was euthanised in MS-222, and then preserved in 100% ethanol. Individuals from one population of the related swamp guppy (*Micropoecilia picta, n* = 5) were also sampled from Trinidad. Fish were collected with written approval from the Director of Fisheries Division, Ministry of Agriculture, Land and Marine Resources, Trinidad and Tobago.

### Molecular methods

DNA was extracted from three to five dried scales or pectoral fins using a glassmilk-binding protocol^61^. Samples were genotyped at 10 polymorphic microsatellite loci using *P. reticulata-*specific primers^62–64^. DNA was amplified via PCR in 5 μl volumes comprising 10–50 ng DNA, 0.5 µl 10× ThermoPol PCR buffer (20 mM Tris-HCl, 10 mM KCl, 10 mM (NH4)2SO_4_, 0.1 % Triton X-100), 200 µM dNTP, 200 µM fluorescently labelled forward primer, 200 μM reverse primer and 0.5U *Taq* DNA polymerase (New England BioLabs). PCR amplification consisted of the following: 4 min at 95 °C, 30 cycles of 30 s at 95 °C, 30 s at locus-specific annealing temperature (Supplementary Table 2), 30 s at 72 °C, and 3 min at 72 °C. PCRs were carried out in Eppendorf Mastercycler ep thermal cyclers. Microsatellite PCR products were visualised by electrophoresis on 8% denaturing polyacrylamide gels run on a LI-COR IR2 DNA analyzer at 50 °C. All gels included positive control samples, redundant samples and a molecular weight size standard ladder. All analyses were conducted in the R statistical package^65^ unless otherwise stated. Microsatellite genotypes were checked using Micro-Checker v.2.2.3^66^. All 10 loci were checked for selection using LOSITAN^67^, which suggested that one of the 10 loci (Pret-46) did not conform to expectations under neutrality. Moreover, inclusion of this locus resulted in inferences of population structure that were bio-geographically implausible and contradictory to those previously reported in guppies^29^, and so was removed.

A 209-base pair (bp) fragment of MHC IIb, encompassing all but three codons predicted to comprise the PBR was amplified using the degenerate primer pair DABdegFb–GTGTCTTTARCTCSHCTGARC^68^, and DABdegRei–CTCACCTGATTTAKYYAG^26^. Each primer was uniquely modified on the 5′end with a 10-bp multiplex identifier (MID; Roche Diagnostics Technical Bulletin TCB No.005–2009), and samples were amplified using a unique combination of forward and reverse MID-labelled primers, which allowed recovery of the amplicons per individual after demultiplexing. PCRs contained 0.2 mM dNTPs (New England Biolabs), 0.5l M forward primer, 0.5l M reverse primer,19 Phusion HF buffer, 6% DMSO,~1–3 ng genomic DNA and 0.4 U Phusion DNA Polymerase (Finnzymes). PCRs were performed in Mastercycler Epgradient S (96-well), or ep384 thermocyclers (Eppendorf), using the following parameters: 98 °C for 3 min; 30 cycles of 98 °C 15 s, 57 °C 40 s,72 °C 60 s; 10 min at 72 °C, then held at10 °C. PCR amplicons were pooled and prepared for 150-bp paired-end Illumina MiSeq (Illumina, Inc., San Diego, CA, USA) sequencing using the vendor’s TruSeq library protocol.

To infer MHC genotypes, we used ultra-deep sequencing and error correction to identify a sequencing break point (or a Degree of Change—DOC) between the cumulative depth distribution of alleles and sequencing artefacts^26^. The approach assumes that sequence errors attain significantly less sequencing depth than true alleles when compared within an amplicon. This approach provided accurate and repeatable genotype estimates of co-amplified loci. The application of ultra-deep sequencing is an effective approach to mitigate the effects of random-allele drop out^69^, where one allele may fail to be efficiently amplified. Indeed, we previously observed very high genotyping repeatability (low allele drop-out) among samples within a sequencing run (100%) and between sequencing runs (83.7%)^26^. Moreover, repeatability in this study rose to 99.83% when samples (including replicate PCRs) were sequenced among four independent Illumina MiSeq sequence runs. This meant that very little random-allele drop out occurred when using the same primers and ultra-deep sequencing as previously described^26^. We also previously showed that this genotyping approach and PCR primers produced no detectable amplification and genotyping bias (which could be impeded genotyping accuracy) even below a total amplicon depth of 100×^26^. We were therefore confident that the population-level variation in the total number of alleles per individual (or gene copy number variation) reflected real biological processes and not genotyping artefact.

### Supertype classification

We grouped MHC IIb alleles into functional supertypes (ST) by analysis of amino acid polymorphism at the guppy-specific peptide-binding region (PBR)^26^. The PBR is the adaptive interface of pathogen recognition leading to the host immune response^70^, and it is under positive selection. Therefore, PBR diversity should reflect functional differences among alleles. The PBR was inferred previously from amino acids that showed an elevated posterior probability of positive selection, based on dN/dS ratios under a Bayesian population genetics framework^26^. The alleles of all three guppy species were pooled for PBR identification, and justified as follows: (1) many alleles were shared between *P. reticulata* and *P. obscura*, (2) these species share the same infecting parasite species, (3) the small number of *M. picta* samples did not allow for a separate analysis, (4) we aimed to identify codons under selection among species, which would allow phylogenetic analysis among homologous codons, and (5) experimentally validated crystallography evidence confirmed shared PBR amino acid sites across taxonomically diverse species, e.g., ref. ^71^. The PBR of each allele was numerically characterised based on the physicochemical properties of each amino acid^19^, based on five metric descriptors: *z1*(hydrophobicity), *z2* (steric bulk), *z3* (polarity), *z4* and *z5* (electronic effects)^72^. We produced a matrix with rows representing each allele and columns representing z1–z5 for each amino acid of the PBR, concatenated in sequence. Using this matrix, alleles were clustered by discriminant analysis of principle components (DAPC) with the *adagenet* package^73, 74^ in R^65^. ST classification avoided introducing missing data points into correlation analyses, where the presence of alleles varied greatly among populations, but the presence of STs was more consistent (see ‘Results’ section). Bayesian information criterion (BIC) values were used to explore different clustering solutions. The optimal number of STs was defined as the minimal number of clusters after which the BIC increases as indicated by BIC values as a function of cluster number^74^. After identifying the optimal number of clusters, we applied DAPC, and the ST clusters were visualised using the first two PCs. To validate the robustness of ST inference, we repeated the analysis for a subset of the samples, including ~50% of the individuals per population (820 in total), comprising 407 alleles (76% of total), which also confirmed 15 STs.

### Population and supertype genetic diversity

Population differentiation was calculated using Jost’s D, which is more appropriate than other statistics given that MHC loci in guppies are highly diverse, exist in multiple copies, and because the locus affiliation of alleles is unknown^75^. This is the case with many MHC population genetic studies and poses an issue when estimating population differentiation, as many software programs require the designation of alleles to loci. The arbitrary allocation of alleles to loci may severely bias population genetic estimates. As such we used custom scripts modified from SPADE R package^76^, which estimates Jost’s D by comparing population-level allelic frequencies (i.e., allele pool unassigned to loci), which is appropriate when estimating population differentiation in MHC data sets. Like *F*_ST_, at complete differentiation, *D*_est_ equals unity, and *D*_est_ equals zero when the allele frequencies of populations are identical. Differentiation was estimated independently using MHC allele, MHC ST, and microsatellite allele frequencies. We tested whether population differentiation estimates based on STs were lower because of simply reducing the amount of diversity being compared among populations (i.e., a random bioinformatic artifact) or reflected a real biological phenomenon driven by balancing selection. Given the observation of 15 STs, we therefore randomly distributed the observed alleles in 15 artificial groups. In this procedure, the MHC allele genotypes remained the same as the observed ones, but each individual now comprised a random ST genotype. We compared estimates of ST population differentiation computed using the empirical data to the random distribution of estimates. To achieve this, code was written which implements the following procedure: For 1000 iterations, (a) clear the ST designations across all alleles in the empirical data, (b) without replacement, randomly reallocate the alleles in the total gene pool to the same number of groups as observed STs, which receive the same number of total alleles as observed in the empirical data. Each individual now comprised an MHC allelic genotype that is unchanged from the empirical data but a random MHC ST genotype, (c) count the number of individual randomised occurrences of each ST present in each population. This results in a matrix of ST counts per population, where each row is a ST, and each column is a population, (d) calculate the pairwise Jost’s D statistic for the matrix generated by step c, and then the average of each column, i.e., compute the mean Jost’s D statistic for each population.

Pairwise values of *D*_est_ were compared among microsatellites, MHC alleles and MHC STs using a Mantel test in the ape package^77^ with 10,000 iterations, and Holm corrected *P* values for multiple comparisons. For each ST, we calculated (1) the total number of MHC alleles (nucleotide variants), (2) the number of PBR amino acid sequences, (3) the mean distance among PBR amino acid sequences (number of differences) within an ST, (4) the mean frequency of an ST in populations, (5) the mean number of alleles within each ST per population, and (6) the level of PBR redundancy among the constitutional MHC alleles within an ST (*S*_r_). *S*_r_ was calculated as the number of alleles within an ST divided by the number of unique PBR amino acid sequences in the same ST. Sequence similarity and dendrograms of MHC alleles and PBR sequences were inferred in MEGA 5^78^, and edited in FIGTREE (www.tree.bio.ed.ac.uk/software/figtree/).

We also analysed whether there was a relative deficiency of individuals with two copies of the same ST (‘homozygote ST’), which would be expected if STs were under balancing selection. Given that 1600 out of 1675 individuals (99%) possessed two or fewer copies of the same ST, we assumed for this analysis that STs are locus specific. Also, we refer to individuals with two copies of the same ST as guppies with a ‘homozygote ST’ genotype. We calculated for each ST the deviation between the observed and expected number of pairwise combinations of ‘homozygote ST’, and we summed this across all populations (*n* = 55). On average, populations possessed 7.96 STs, so the total number of comparisons was 55 × 7.96 = 438. The deviation from zero was tested using a paired *t*-test.

### Linkage disequilibrium and Hardy–Weinberg equilibrium

Because locus affiliations of MHC alleles and STs are not known when multiple duplicated loci are co-amplified using degenerate primers, we were unable to use published software that calculates linkage disequilibrium (LD) between pairwise combinations of loci. Therefore, the relative excess of associations between certain STs within individual genotypes was analysed to examine evidence of LD, which is a statistically robust approach to detect patterns of association. LD (i.e., an excess of observed combinations between certain STs relative to the expected count) is indicative of physical linkage between loci on haploblocks, although demographic effects and cryptic population substructure can also create significant LD. Furthermore, balancing selection acting on two (or more) loci will also reinforce LD through epistasis, as increased LD would reduce the segregation load. In our LD analysis, we calculated the deviation between the observed and expected frequencies of all possible pairs of STs within individuals of a population. Given that we identified 15 STs, the maximum number of pairwise comparisons of two STs equals 105 (*N* = (15 × 14)/2 = 105), although this number was generally smaller because not all STs were present in each population. To calculate the observed frequency of a ST pair, all pairwise combinations of those two STs within an individual were counted, and this was summed across all individuals within the population. This value was then divided by the total number of pairwise combinations of STs within all individuals of that population. The expected frequency of a ST pair was calculated by first establishing the frequencies of the two STs in the population, and then calculating the product of the two ST frequencies. Finally, the deviation between the observed and expected frequency of each ST pair was tested using a paired *t*-test. The *P* value was corrected for multiple comparison using a sequential Bonferroni correction. The deviations between the observed and expected frequencies were visualised in XY-graphs, with the expected frequency of the ST pair on the *X* axis and the observed value on the *Y* axis. Values above the line *X* = *Y* indicate a relative excess (and hence, LD), and values below this line a shortage of the ST pair in a population (consistent with repulsion).

### Computer simulations of immune gene evolution

We developed an agent-based model to study the evolution of immune genes in a host–parasite system, examining whether TSP of STs can evolve in a Red Queen arms race. Rather than using a strict population genetic model, in which alleles and genotypes are assigned fitness values, this model was based on ‘epitope space theory’^24, 25^ that supports a finite epitope space in which parasite antigens and host immune recognition molecules co-evolve. We analysed the adaptive evolutionary change in epitope recognition of immune alleles and STs (i.e., their paratope) during host–parasite co-evolution. We aimed to construct the most basic model that (1) would result in antagonistic host–parasite co-evolution, and (2) in which we could quantify the resulting adaptive evolutionary change in phenotype over time. Hence, rather than using a strict population genetic model, we modelled the paratope of immune alleles and the epitope of parasites in a 2D grid with size 1000 × 1000, which fits with current antigen/epitope modelling theory^24, 25^. The relative position of each immune allele and the parasites in this space thus determines the selection coefficient acting on each immune allele, and the fitness of an individual is proportional to the Euclidian distance between the antigen and immune allele in the epitope/paratope space. The adaptive evolutionary change in phenotype of alleles and STs was quantified by tracking changes in their position within this space over a large period of evolutionary time. Analysing the phenotypic change enabled us to study trans-species polymorphism (TSP). Furthermore, by analysing fluctuations in immune allele frequencies, we could study the population genetic characteristics of the model.

The simulation began with both host and parasite alleles randomly distributed across the epitope/paratopes space. Hosts were diploid with one or three immune loci. In the main text, we show the results of a single locus model, and in Supplementary Fig. 12 we show a 3-locus model without recombination (i.e., loci were completely linked). Parasites were haploid, and each host was infected by one parasite every generation. The minimum Euclidean distance was calculated between an individual’s immune alleles and one randomly drawn parasite representing the infection. Depending on this distance, the parasite was either recognised (in a resistant host) or not (in a susceptible host). Fitness was relative so that 50% of all parasites died (on resistant hosts). The other 50% of parasites (on susceptible hosts) reproduced clonally one individual offspring. The epitope of parasite offspring mutated, causing a change in *X* or *Y* coordinates by one unit within the grid. Parasite infection on the susceptible hosts reduced host fitness by 0.25. (Hence, host generation time was ≥4 times longer than the parasite generation time). Host with zero fitness died. Resistant host gained 0.25 fitness units, and individuals with one fitness units reproduced offspring that all started with 0.25 fitness units. Hosts reproduced sexually, producing gametes with one parental immune allele each. This immune allele mutated with probability *µ*, which caused it to change its *X* or *Y* coordinates by one unit within the grid. Shown are the results with a high mutation rate (*µ* = 0.1), which effectively accelerates evolutionary time in the model. With *µ* = 0.1, the evolutionary time is accelerated by a factor 3.1 × 10^6^, assuming a base mutation rate of 10^−9^ per base per generation, and 16 PBR codons with a total of 32 replacement sites (i.e., the first and second codon positions of the PBR). Gametes of reproducing hosts united randomly to produce the next generation of diploid offspring. This resulted in a Poisson distribution of offspring per parent (mean = variance = unity).

Alleles were individually labelled at the start of the simulations, which enabled us to track the ancestry of extant alleles and define STs. In the model, an ST is defined as all the alleles that belonged to the same ancestral allele that was identified by its unique label, i.e., an ST is a group of alleles that coalesced with each other at the start of the simulations. We opted for this approach in our simplified model because without recombination between alleles, the ancestry of alleles in our model is unambivalent, which enabled us to identify STs. The position of alleles and parasites in the grid was recorded every time step, and the centroid of an ST was found by calculating the mean *X* and *Y* coordinate of the alleles belonging to that ST. We examined the evidence of TSP by analysing the rate of adaptive evolutionary change (i.e., change in the position of centroid) of STs over time. For each ST, its nearest neighbour (after the burn-in at generation *t* = 1000) was determined. To study the population genetics of host–parasite co-evolution, we also recorded the changes in frequency of immune alleles of one ST over time. Finally, to study the effects of drift on allele and ST variation, we analysed the effect of population bottlenecks, simulating host population sizes *N* = 10^4^, 10^3^ and 10^2^, and recording the number of alleles and STs present in the host population.

In nature, the epitope space faced by a host population is vast and constantly changing, and in order to make it more tractable, we simulated a large but finite epitope space, which is consistent with the current understanding of the antigen/epitope modelling theory^24, 25^. Setting boundaries does not generate a ‘magic number’ of stable STs; simulations show that this number depends on the strength of parasite selection, the amount of genetic drift (and effective population size), as well as the number of MHC loci simulated. This is also consistent with DAA^21^ and MHC evolution in nature; ST variation of HLA-B differs geographically among (human) populations, suggesting that selection on STs reflects local adaptation to different parasite communities^47^. In addition, we did not simulate multiple parasite infections, and therefore, natural systems may demonstrate more complex interactions among host and parasite communities than simulated here.

### Ethical approval

All applicable international, national and/or institutional guidelines for the care and use of animals were followed.

### Code availability

The code for host–parasite co-evolutionary simulations was developed in Minitab and it is available from GitHub along with R scripts to perform ST analyses (https://github.com/Ward9250/Supertypes_RedQueen_TSE).

### Data availability

The data sets generated during and/or analysed during the current study are available in the NCBI database (www.ncbi.nlm.nih.gov) in the form of MHC allelic sequences: accessions KF321642.1–KF321728.1 (PopSet: 544451456), and KT003989.1–KT004363.1 (PopSet: 1033321404). All other data generated or analysed during this study are included in this published article (and its supplementary information files).

## Electronic supplementary material

Supplementary Information

Description of Additional Supplementary Files

Supplementary Dataset 1

Supplementary Dataset 2

Supplementary Dataset 3

Supplementary Dataset 4

Supplementary Dataset 5

Supplementary Dataset 6

Supplementary Dataset 7

Supplementary Dataset 8
