# Supplementary material for: Evolutionary genetics of immunological supertypes reveals two faces of the Red Queen
Source: Nat Commun. 2017 Nov 3;8:1294. doi: 10.1038/s41467-017-01183-2 (PMC5670221; doi:10.1038/s41467-017-01183-2)
Supplement: Supplementary file 1 — Supplementary Information [file 41467_2017_1183_MOESM1_ESM.pdf]

Supplementary Figures and Tables

(a)

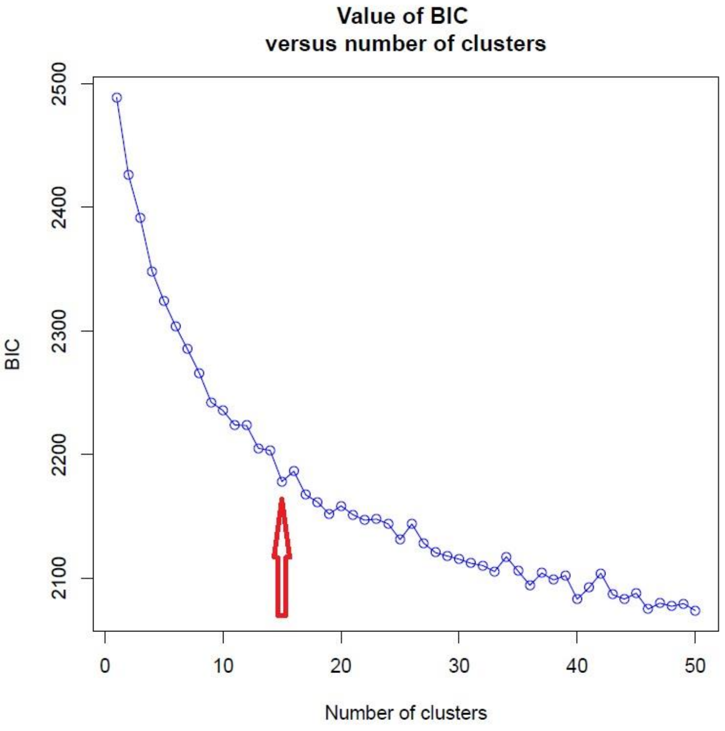

(b)

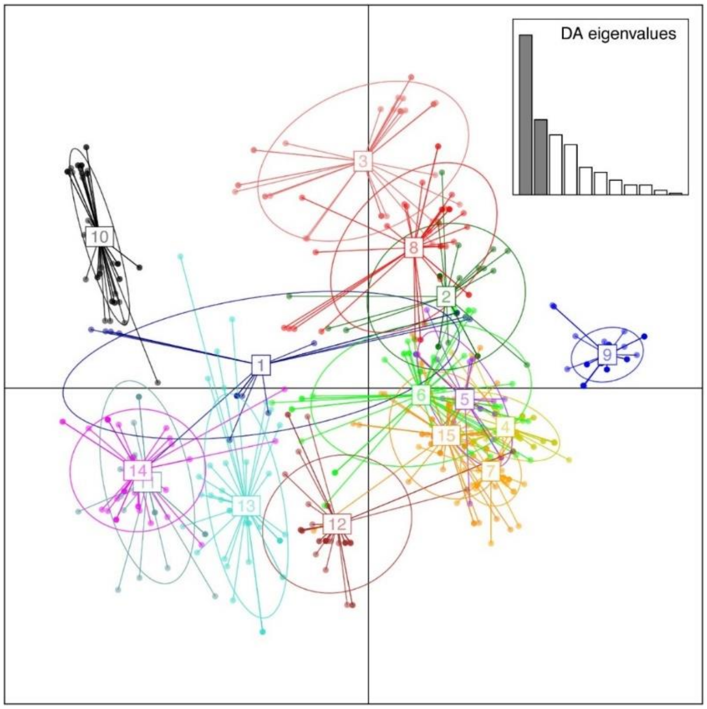

(c)

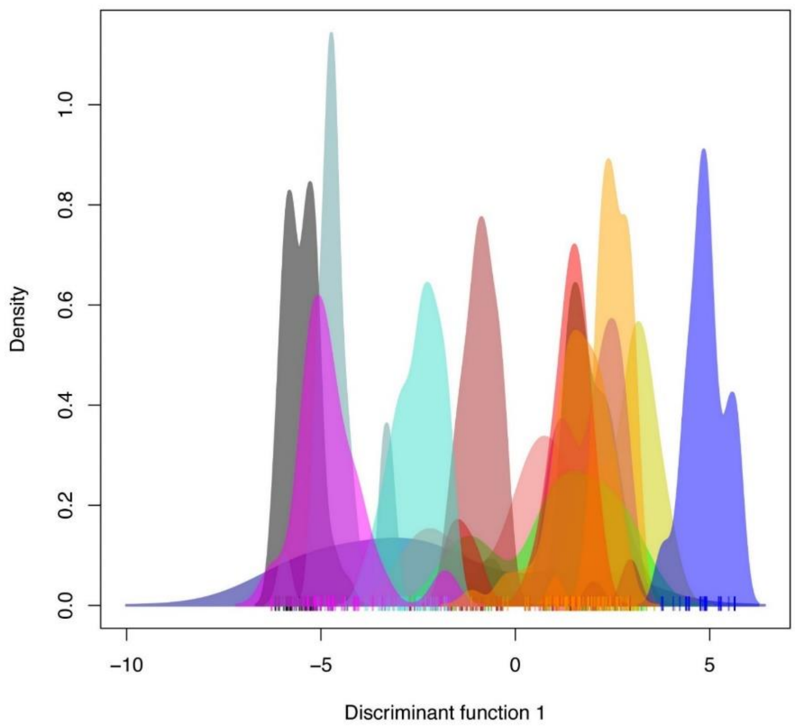
Density
0.0
0.2
0.4
0.6
0.8
1.0
−10
−5
0
5
Discriminant function 1

(d)

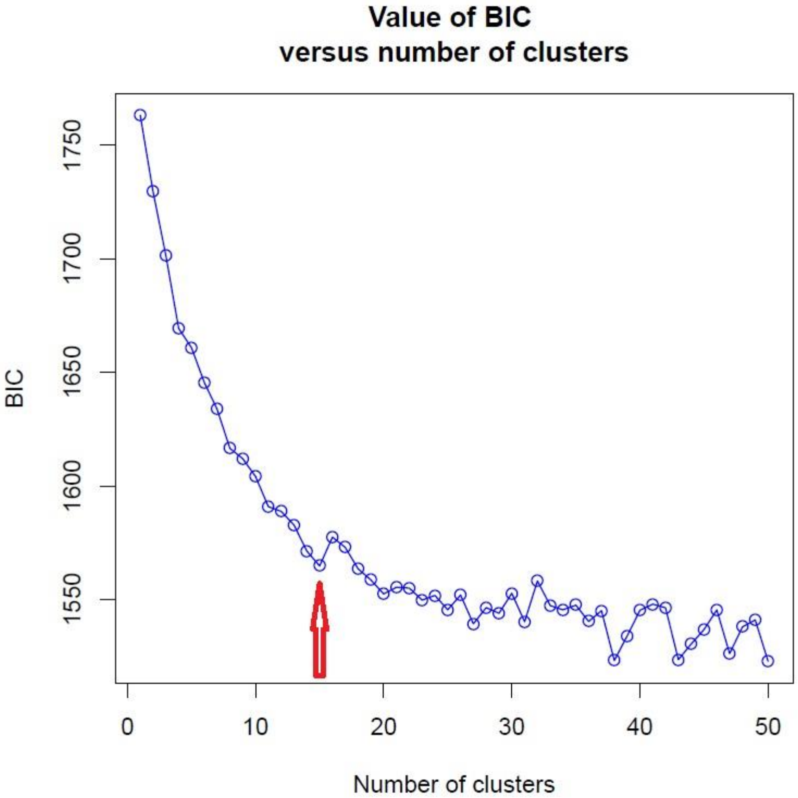
Value of BIC
versus number of clusters
BIC
1550
1600
1650
1700
1750
0
10
20
30
40
50
Number of clusters

Supplementary Figure 1. Clustering of MHC alleles by Discriminant Analysis of Principle Components (DAPC), based on the physicochemical properties of translated amino acids inferred to comprise the Peptide Binding Region (PBR). (a) DAPC showed the presence of fifteen clusters (supertypes) which is inferred by the first increase (or elbow) in Bayesian Information Criterion relative to iterations of clustering[6] (red arrow), which are visualized on (b) two, and (c) one discriminant function. (d) DAPC based on a randomly drawn subset of the data representing approximately fifty per cent of the individuals from each population and a total of 407 alleles (76% of total); the analysis corroborates the findings based on the total dataset, confirming the robustness of the initial estimate of 15 supertypes.

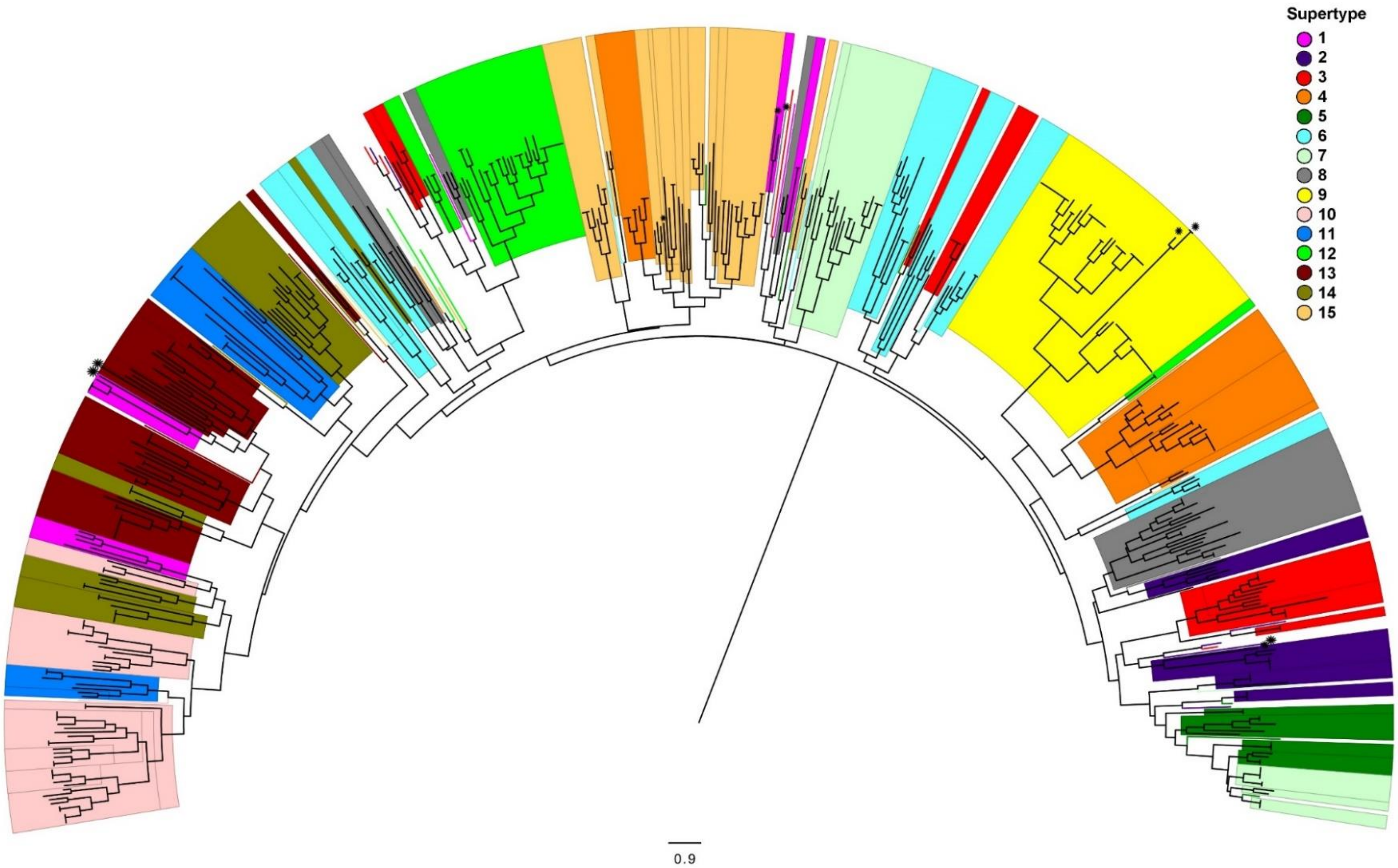

Supplementary Figure 2. Neighbor-joining dendrogram of relationships among MHC alleles, based on the translated amino acids inferred to comprise the Peptide Binding Region (PBR). For clarity, allele names have been removed. Alleles are highlighted based on inferred membership to the 15 supertypes (STs) inferred though by Discriminant Analysis of Principle Components (DAPC). Perpendicular branch tips highlight identical PBR sequences derived from disparate MHC alleles (nucleotide sequences). This analysis shows that except for ST-9, STs are not monophyletic. This suggests that gene conversion events between alleles of different STs contributes to the nucleotide variation within STs. Evidence for such gene conversion has been reported in other MHC studies, including the Berthelot's pipit (*Anthus berthelotii*), where the number of novel haplotypes generated by gene conversion across divergent lineages exceeded the rate of point mutation by an order of magnitude[1]. The 10 alleles observed in five *Micropoecilia picta* samples are denoted by asterisks.

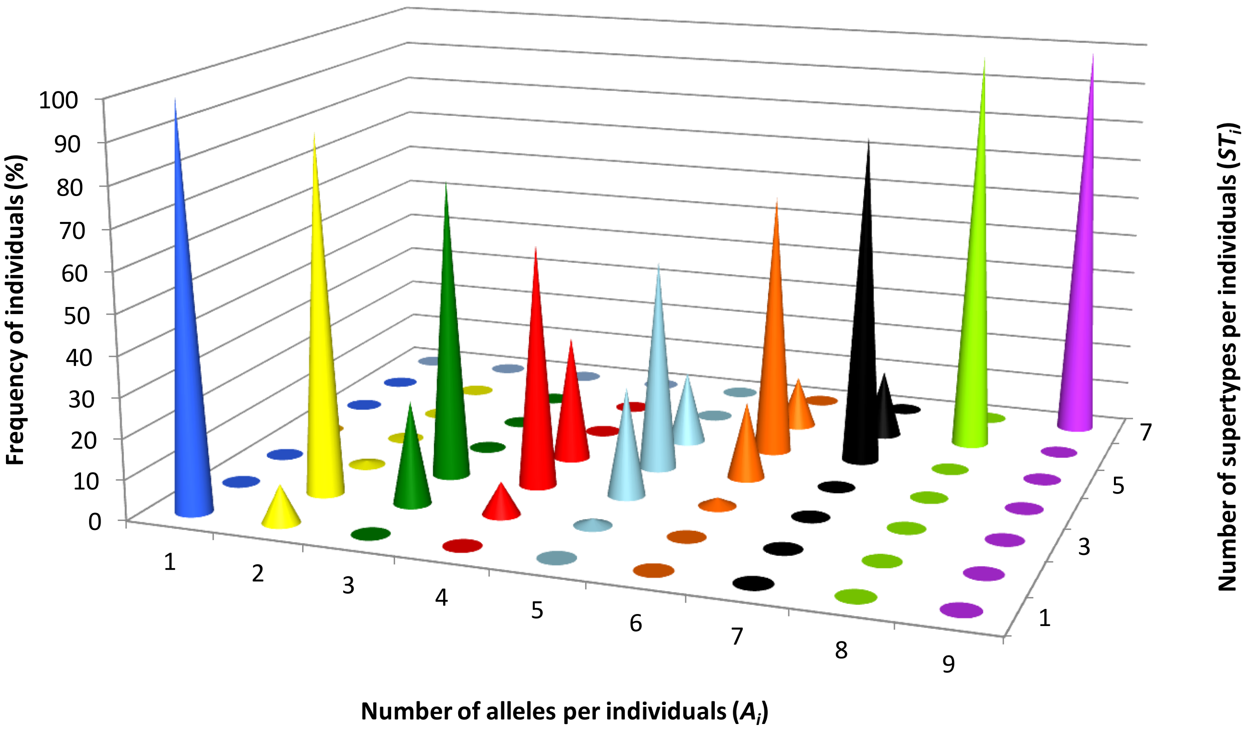

Supplementary Figure 3. The relationship between the number of alleles within an individual (A_i) and the number of supertypes (STs) within an individual (ST_i) observed among all samples. In general, as A_i increases, so does ST_i; however, there is variability in the trend, with intermediate levels of A_i (A_i = 4-6) corresponding to more variation in ST_i.

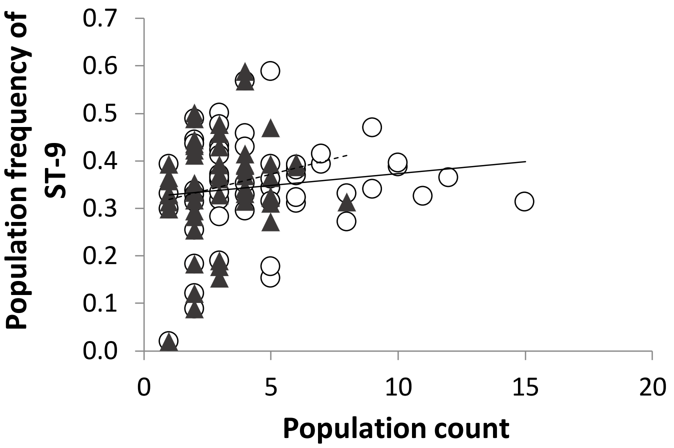

Supplementary Figure 4. The lack of relationship between the number of unique alleles (open circles) and cumulative population frequency of supertype-9 (ST-9) (Linear regression, solid line; *P*=0.303, R^2=0.01), as well as between the number of unique PBR sequences (solid triangles) within a population, and the combined frequency of ST-9 alleles (Linear regression, hashed line; *P*=0.162, R^2=0.03) present in a population again supports strong balancing selection on ST frequency.

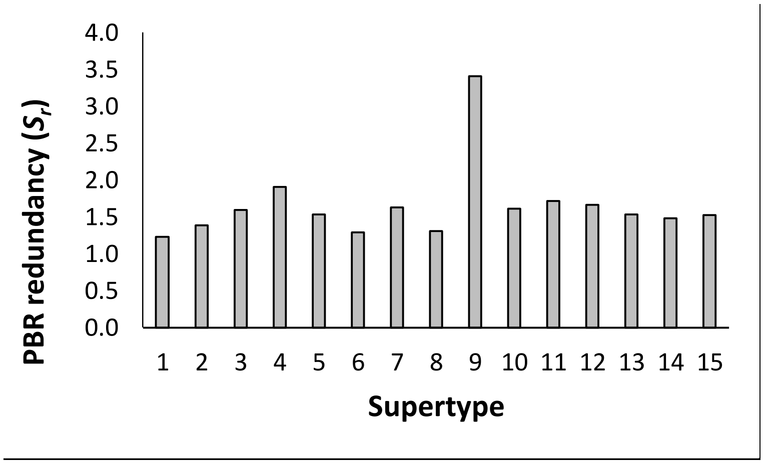

Supplementary Figure 5. Redundancy of the Peptide Binding Region (PBR) translated from the unique MHC allelic nucleotide sequences in each supertype (ST) (S_r). Redundancy increases as the fraction of unique PBR sequences decreases among alleles within a ST. In general, the majority of STs display a similar degree of PBR redundancy, while ST-9 displays almost double the amount of PBR redundancy compared to all other STs. This high redundancy is coupled with the highest number of MHC alleles of any ST. In conjunction with the near ubiquitous nature of ST-9, this suggests that its functionality has been preserved over large temporal and spatial scales, expanding in the number of alleles through mutations that do not deviate from the functionality that is under strong balancing selection.

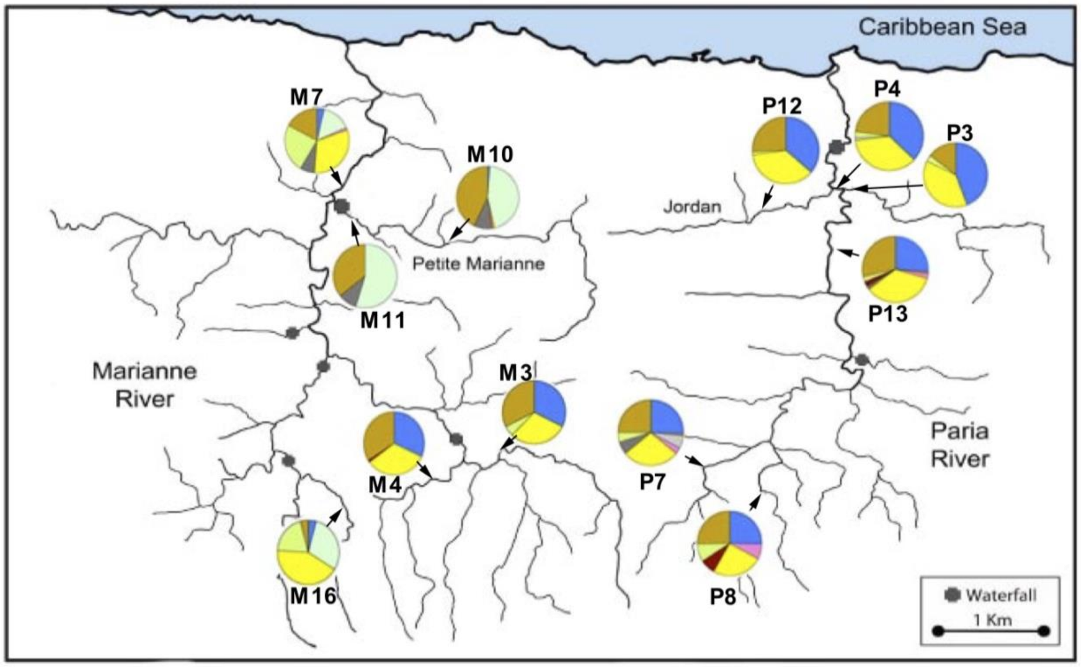

Supplementary Figure 6. The geographic distribution of MHC supertypes (STs) in guppy populations across Trinidad and other islands (See Fig 3 for full details). Abbreviated populations comprise; Diego Martin (DM), Curaguate (Cur), Las Cuevas (LC), Yarra 1 (Y1), Yarra (Y2), Damier 1 (D1), Damier (D2), Marianne 3, 4, 7, 10, 11, 16 (M3, M4, M7, M10, M11, M16), Paria 3, 4, 7, 8 ,12, 13 (P3, P4, P7, P8, P12, P13) Madamas (Mad), Shark (Sh), San Souci (SS), Mission (Mis), Tompire (Tom), Cumana (Cu), Matura (Mat), Las Seiva (LS), Turure 2 (T2), Turure 3 (T3), Quare 1 (Q1), Quare 2 (Q2), Aripo 1 (Ap1), Aripo 2 (Ap2), Guanapo 1 (G1), Guanapo 2 (G2), El Cedro 1 (EC1), El Cedro 2 (EC2), Arima 1 (Ar 1), Arima 2 (Ar2), Silver Stream (Sil), Pitch Lake (PL), Point Fortin (PF1), Point Fortin 2 (PF2), SN (Snake), Stollmeyer (Sm), Vance (Vc), Morne (Mn), Mondesir (Mon), Fifth Company (FC), Oropouche-South (OS), Basse Terre (BS), Poole (Po), Faecal Dump Rd. (FDR), Guayguayre (Gg). Guppies were also collected from Oahu and Maui in the Hawaiian archipelago, Windward Rd. in Tobago, and Grahame Hall swamp in Barbados. *Micropoecilia picta* were collected from St. Joseph, East coast Trinidad. Complex variation in ST frequency distribution occurs across populations, and for clarity those in the Marianne and Paria are shown here separately. ST9 is obviously maintained in similar frequencies across the majority of populations, despite wide variation in the frequencies of particular alleles that are present. Populations that are in close proximity tend to be similar in ST composition, which is likely a consequence of similar selection pressures and gene flow. For example, Cumana is fixed for just one ST-allele that is found in the neighboring Tompire River (and Pitch Lake in the South West). Populations in the Yarra, Damier, Marianne, and Paria also appear to be largely similar. Damier populations represent a human mediated introduction from the Yarra, but the similarity among (B) the eastern upland populations of the Marianne and those in the Paria likely represent recent colonization of the Paria from the Marianne, and microsatellite data support this[2]. Populations M10 and M11 lack ST9, but are unique in displaying a large expansion of ST4 alleles, which are rare elsewhere.

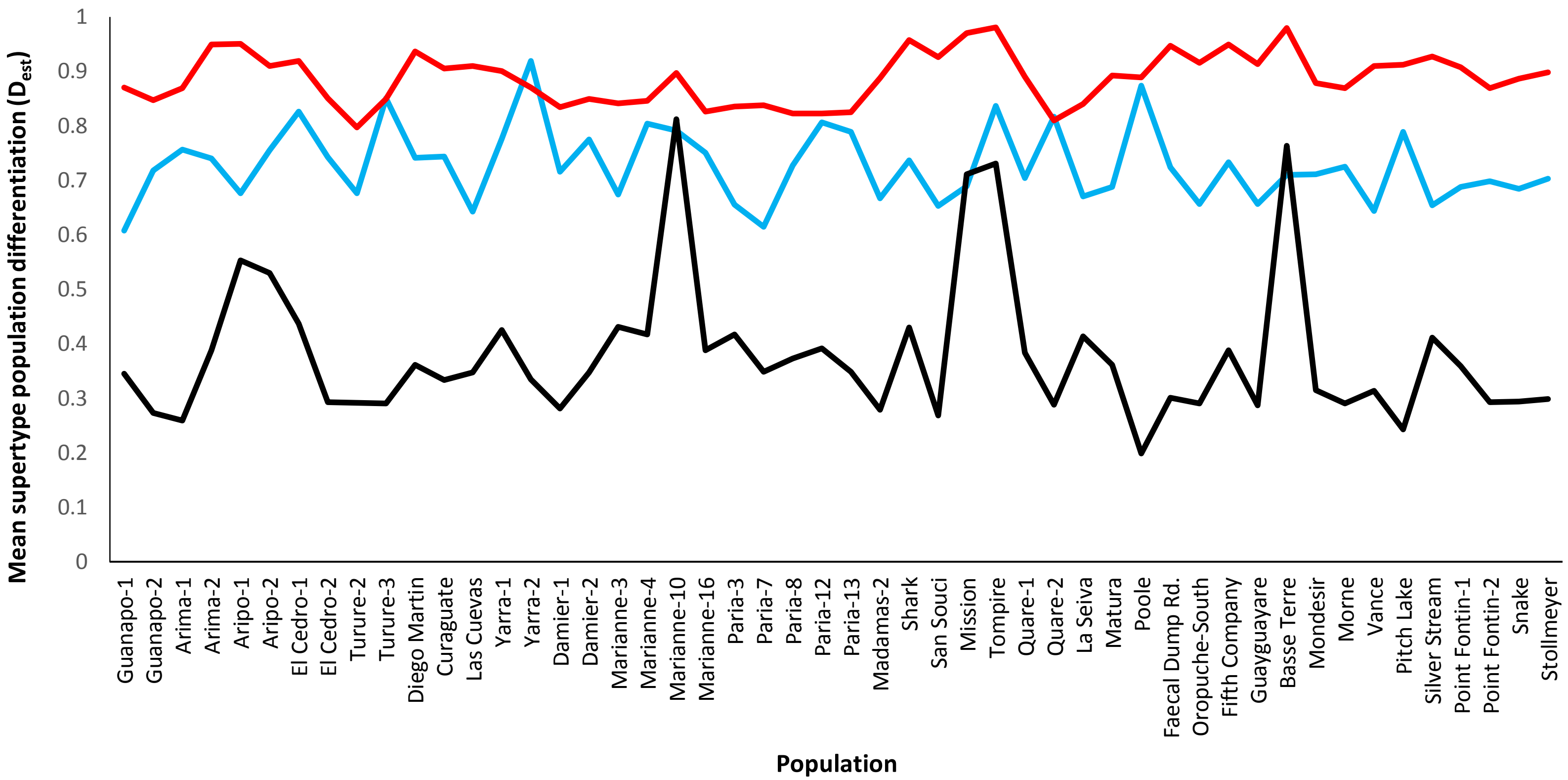

Supplementary Figure 7. Comparisons of mean population differentiation estimates (Jost's D) based on microsatellite (Blue), MHC allele (Red), and MHC supertypes (ST) (Black) frequencies. Although populations are independent, measure of differentiation among genetic data are compared using a line chart for clarity. Generally, guppy populations are highly differentiated based on MHC allele frequencies, as well as in microsatellite allele frequencies, and these are significantly correlated. While most populations are only moderately differentiated based on ST frequencies, representing balancing selection, a few populations show higher differentiation like that observed in microsatellites. This indicated that although ST and microsatellite frequencies are not correlated across the data set (See Results), that in some cases either ST diversity within a population can be affected by demographic processes or local adaptation results populations becoming more differentiated in their MHC functional repertoire. Examination of MHC alleles and microsatellites alone would lead to the erroneous conclusion that strong divergent selection is operating in concert with demographic processes driving high population differentiation in MHC alleles.

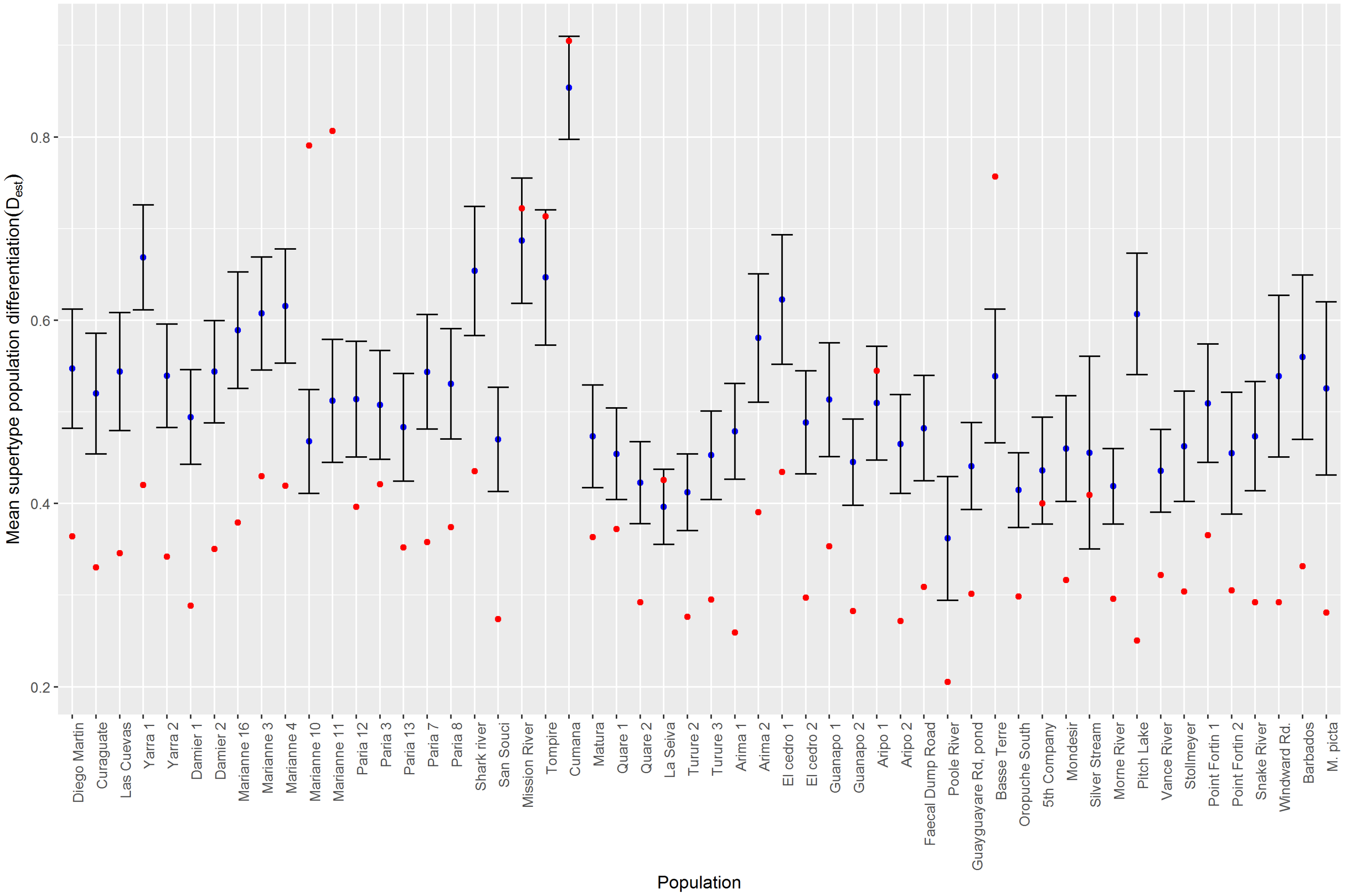
Mean supertype population differentiation(Dest)
0.8
0.6
0.4
0.2
Diego Martin
Curaguate
Las Cuevas
Yarra 1
Yarra 2
Damier 1
Damier 2
Marianne 16
Marianne 3
Marianne 4
Marianne 10
Marianne 11
Paria 12
Paria 3
Paria 13
Paria 7
Paria 8
Shark river
San Souci
Mission River
Tompire
Cumana
Matura
Quare 1
Quare 2
La Seiva
Turure 2
Turure 3
Arima 1
Arima 2
El cedro 1
El cedro 2
Guanapo 1
Guanapo 2
Aripo 1
Aripo 2
Faecal Dump Road
Poole River
Guayguayare Rd, pond
Basse Terre
Oropuche South
5th Company
Mondesir
Silver Stream
Morne River
Pitch Lake
Vance River
Stollmeyer
Point Fortin 1
Point Fortin 2
Snake River
Windward Rd.
Barbados
M. picta
Population

Supplementary Figure 8. Comparisons of mean population differentiation estimates (Jost's D) based on supertypes (STs) estimated from empirical data (Red circles) and simulated data (Blue circles). Simulated mean of D_{est} of each population and 95% confidence intervals were obtained by randomly allocating alleles from the entire data set to STs, which contained the same number of total alleles as estimated from the empirical genotypes. Only seven populations (12.7%) displayed a mean D_{est} that fell within the 95% CI of the randomly simulated estimates of population differentiation, which supports the argument that the level of population differentiation based of ST diversity reflects real biological phenomenon and are not simply an outcome of high-level aggregation of genetic diversity and bioinformatic artifact. Even patterns of ST diversity in populations that fall within simulated distributions may reflect real processes. For example, Cumana consisted of a single allele which was also observed in the close by Tompire River in a homozygous state, and so may represent a founder colonization event.

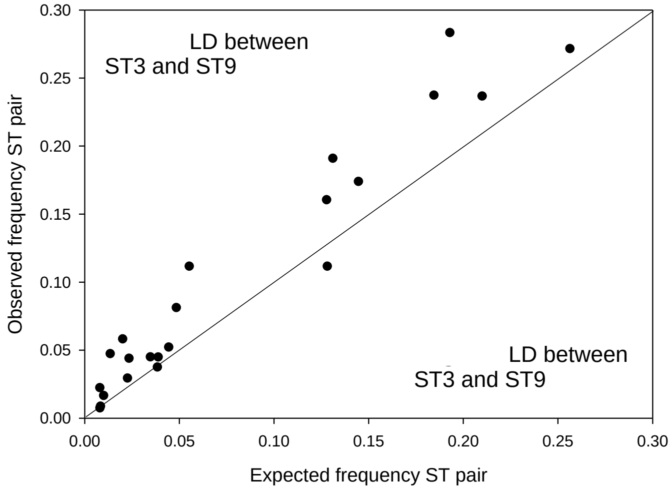
LD between
ST3 and ST9
LD between
ST3 and ST9
Observed frequency ST pair
Expected frequency ST pair
0.00
0.05
0.10
0.15
0.20
0.25
0.30

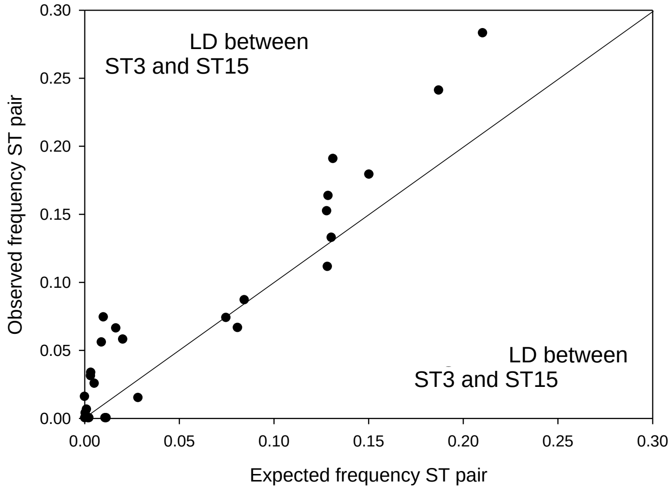
LD between
ST3 and ST15
LD between
ST3 and ST15
Observed frequency ST pair
Expected frequency ST pair
0.00
0.05
0.10
0.15
0.20
0.25
0.30

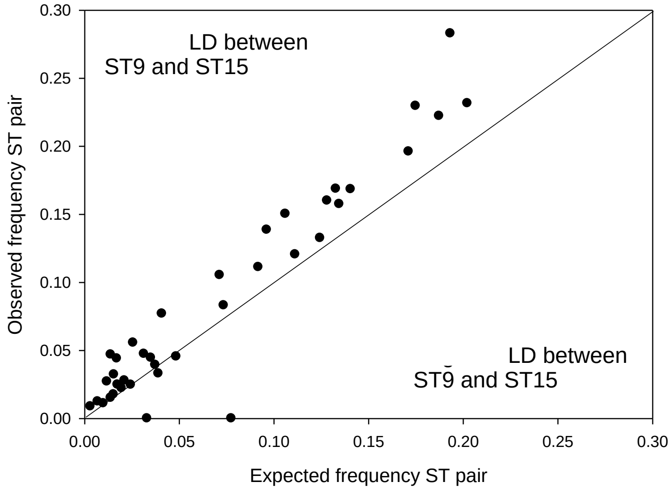
LD between
ST9 and ST15
LD between
ST9 and ST15
Observed frequency ST pair
Expected frequency ST pair
0.00
0.05
0.10
0.15
0.20
0.25
0.30

Supplementary Figure 9. Examples of linkage disequilibrium (LD) between ST3, 9 and 15 are evident by the relative excess of observed pairwise combinations of alleles of these STs in individuals of 55 *P. reticulata, P. obscura* and *M. picta* populations. The LD suggests that the three loci baring ST3, 9 and 15 reside on the same linkage block. Test results of all paired T-tests are displayed in Table S4.1.

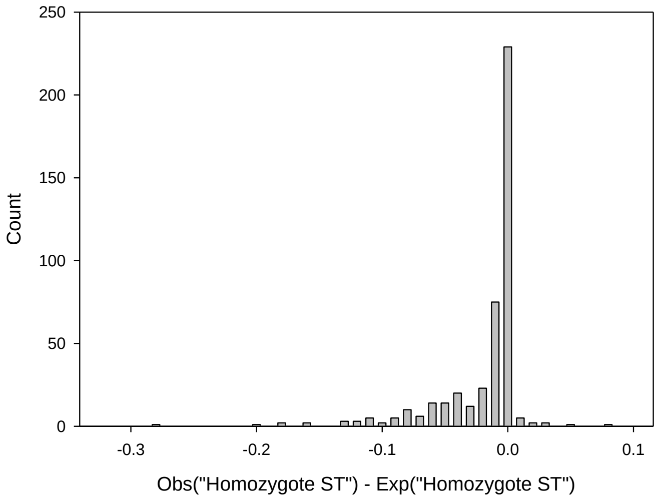

Supplementary Figure 10. Histogram showing the deviation between the observed and expected number of "homozygote ST" individuals for each ST, and summed across all populations (n=438 comparisons in total). The figure shows a large shoulder on the left hand side of zero (observed("homozygote ST") = expected("homozygote ST")). This indicates there are many STs in populations that show a deficiency of individuals with two copies of the same ST, i.e. "homozygote ST" individuals (one sample T-test; T=-10.98, p<0.0001). This implies there is balancing selection acting against individuals with redundant epitope recognition.

.

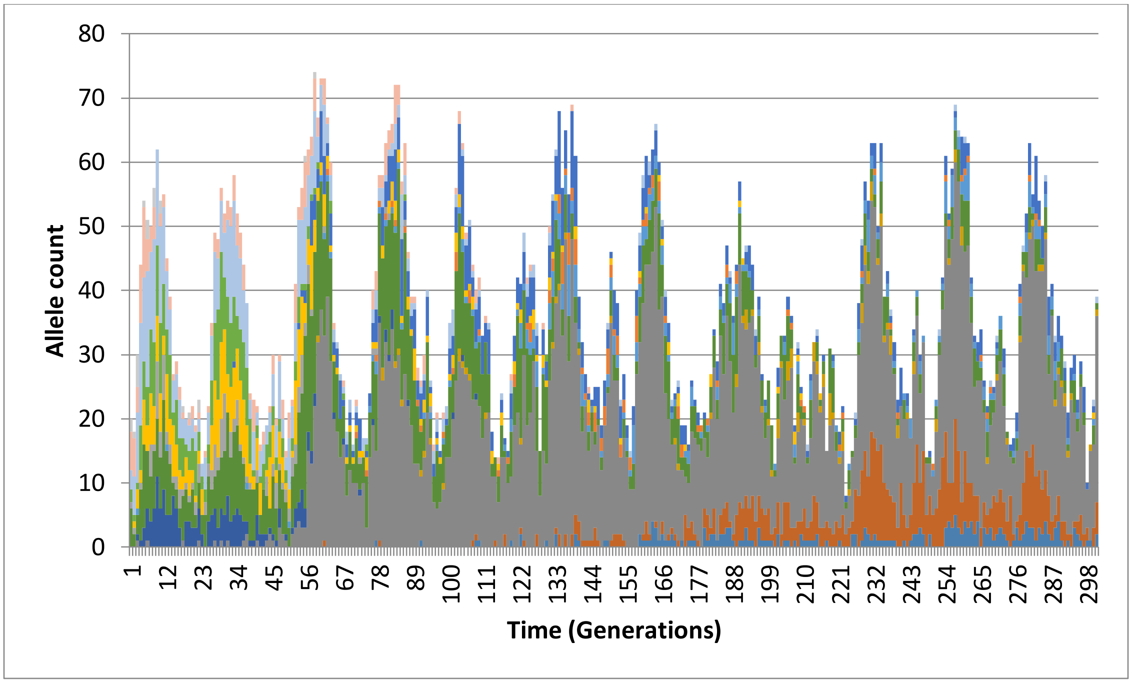

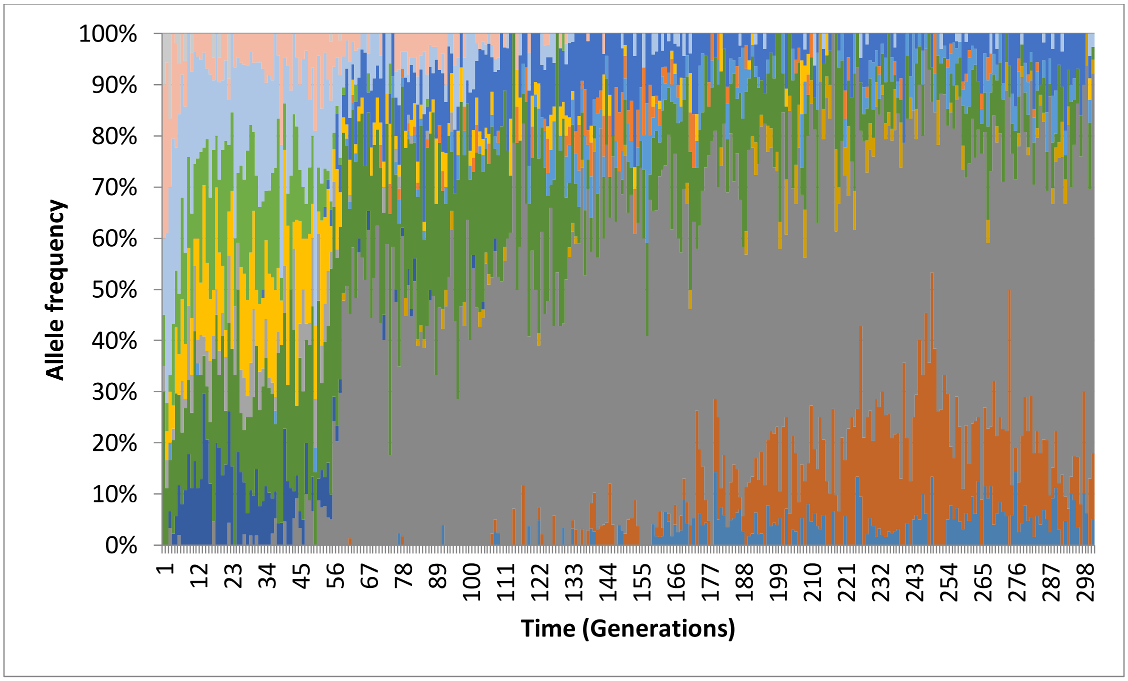

Supplementary Figure 11. Allele frequencies expressed as counts (top) and frequencies (bottom) shows that that the dynamic frequency fluctuations characteristic of the Red Queen arms race are composed of recurrent selective sweeps.

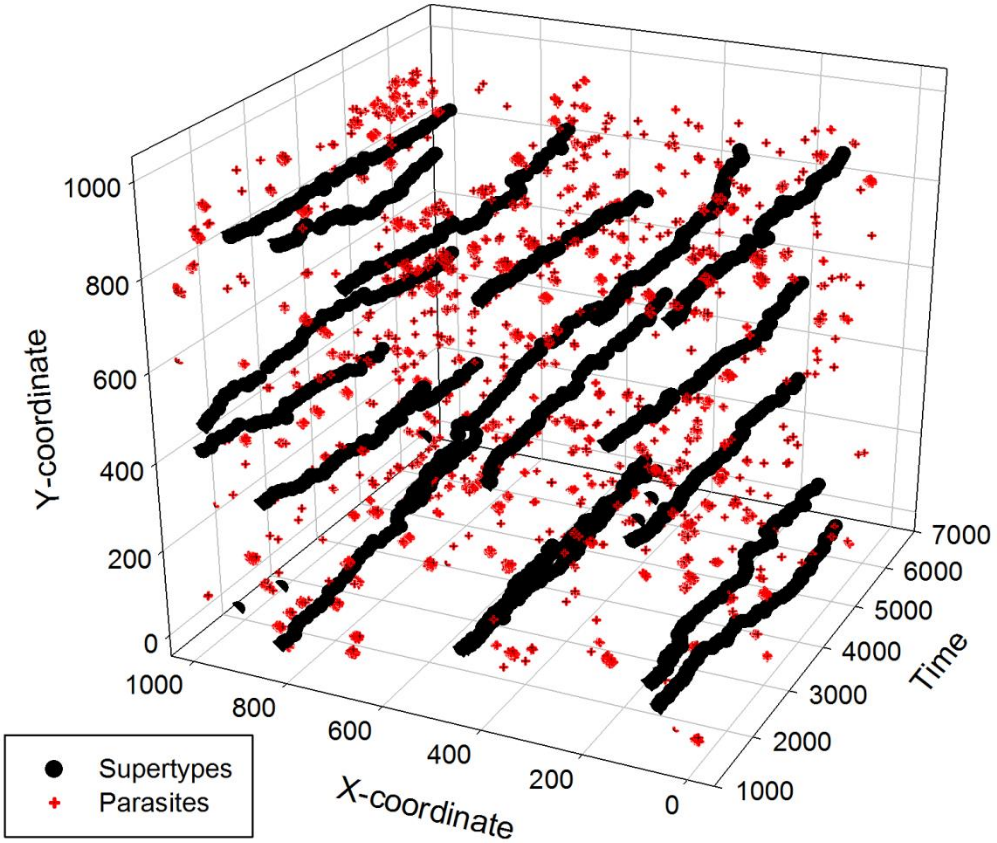

Supplementary Figure 12. Computer simulations of host parasite co-evolution Hosts are diploid with three completely linked immune loci, the host population size equals N=10,000, and mutation rate equals μ=0.1. The centroid position of each supertype (ST) is indicated by the black dots. Parasites are shown as red crosses. In these simulations, 16 STs evolve that remain stable over evolutionary time.

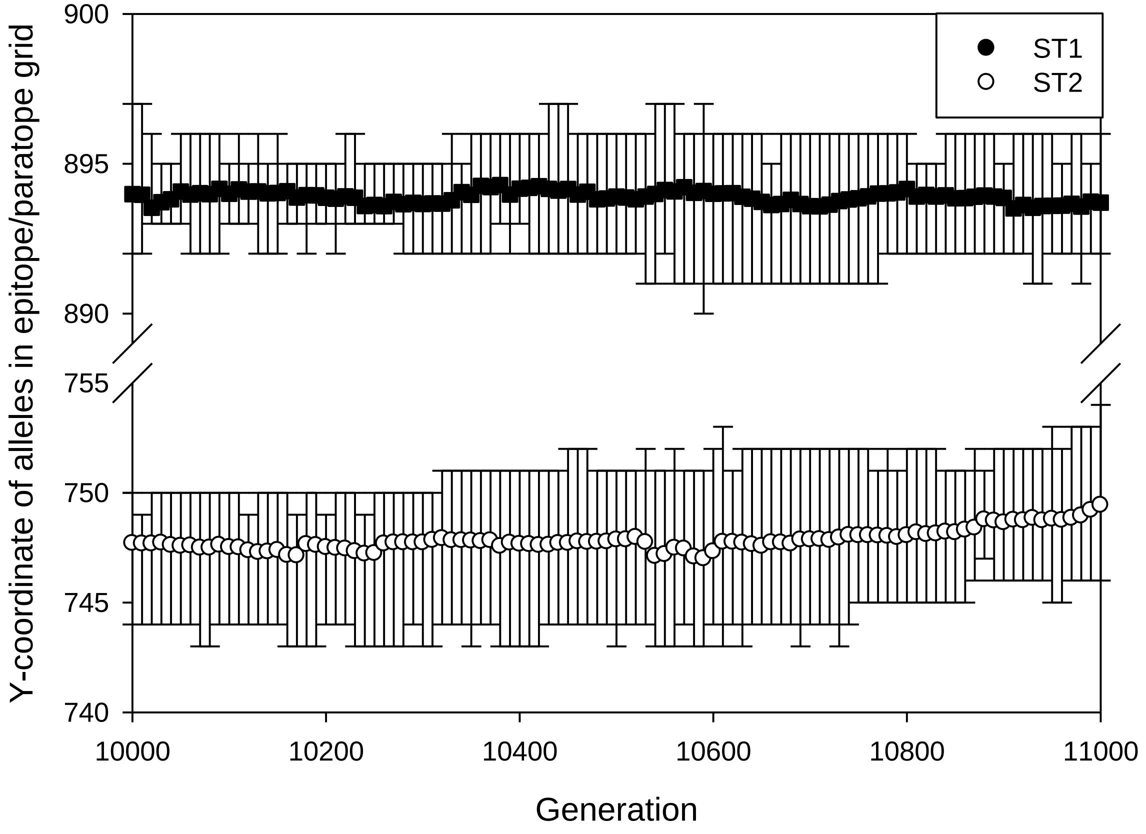

Supplementary Figure 13. The mean and range of the Y-coordinate in the epitope/paratope space of the alleles belonging to ST1 and ST2 in simulations of a population with N=10,000, a single locus, and mutation rate equals μ=0.1. Note that the centroid position (the mean) is relatively stable over time, and that the range of the alleles (error bars) is relatively constant.

Supplementary Table 1. Microsatellite primers used in this study. Listed are the forward and reverse sequences, repeat motif, optimal PCR annealing temperature, GenBank accession number, and source publication for each of the ten primers used.

Locus	***Primer Sequence (5'-3')*** ***F: forward, R: reverse***	***Repeat Sequence***	***Optimal T_A***	***GenBank #***	***Source Publication***
Pre9	**F:** TTGCAAGTCAGTTGATGGTTG<br>**R:** TGCCCTAGGGATGAGAAAAG	$(CAGA)_{13}$	60°C	AY830941	[3]
Pre13	**F:** ACAGTACTGTCTGTCTGTCT<br>**R:** TGTTTGAGACACTCATGGTGAAG	$(CTGT)_{18}$	65°C	AY830942	[3]
Pre15	**F:** CTGAGGGACCAGGATGTTAAG<br>**R:** CCATAAACACGCAAACCAAC	$(GATG)_{16}$	65°C	AY830943	[3]
Pre26	**F:** GCTGACCCCAGAAAAGTGG<br>**R:** TGGGACTTTCATGAGACTTGG	$(GATG)_{19}$	60°C	AY830946	[3]
Pret-27	**F:** CACACGGGCTCTCATTTTT<br>**R:** CTGTGTTTGTGTTCGGTCGTA	$(GT)_{53}$	60°C	AB100321	[4]
Pret-28	**F:** ACATCGGCGTCCTCACCT<br>**R:** GGGGGTTGAAACACATCCA	$(GT)_{32}$	60°C	AB100322	[4]
Pret-38	**F:** AGGGAAAAGGAAAGAAAGAA<br>**R:** CGAACAAGCCCAAATCTA	$(GT)_{19}$	50°C	AB100328	[4]
Pret-80	**F:** GGAAGGGAGGGGAGGAT<br>**R:** CACTTCAGCAGGGCAGACTA	$(GT)_{14}(GA)_{11}$	60°C	AB100354	[4]
G145	**F:** TCTCCAAACCTCCCCTGTA<br>**R:** GACGAGCCTCTGCTTCTTC	$(GT)_{11}$	60°C	DQ855588	[5]

Supplementary Table 2. Summary of the one sample T-test between all pairwise combinations of STs. Combinations that showed significant LD (positive T-value_ or repulsion (negative T-value) after Bonferroni correction are highlighted.

ST locus 1	ST locus 2	T-value	P-value
1	2	-2.13	0.059
1	3	-0.02	0.987
1	4	0.84	0.415
1	5	-2.69	0.043
1	6	0.79	0.441
1	7	-1.33	0.217
1	8	0.42	0.678
1	9	0.9	0.381
1	10	-4.59	0.001
1	11	-0.92	0.374
1	12	-2.47	0.027
1	13	0.68	0.509
1	14	-0.87	0.407
1	15	-0.38	0.711
2	3	0.71	0.498
2	4	-0.58	0.574
2	5	1.38	0.209
2	6	-0.06	0.951
2	7	-0.62	0.55
2	8	0.37	0.716
2	9	1.52	0.144
2	10	1.97	0.067
2	11	-0.53	0.606
2	12	-1.17	0.26
2	13	-0.36	0.725
2	14	-0.37	0.72
2	15	0.19	0.855
3	4	1.27	0.229
3	5	-0.5	0.629
3	6	0.36	0.721
3	7	-0.05	0.963
3	8	0	0.998
3	9	3.64	0.001
3	10	0.56	0.581
3	11	1.41	0.176
3	12	-0.52	0.614
3	13	-0.88	0.39
3	14	-1.13	0.283
3	15	3.43	0.002

4	5	-0.53	0.611
4	6	0.81	0.428
4	7	-1.14	0.298
4	8	-2.44	0.028
4	9	1.77	0.092
4	10	1.49	0.155
4	11	2.07	0.055
4	12	-1.54	0.142
4	13	-0.62	0.543
4	14	-1.45	0.182
4	15	-1.03	0.316
5	6	-2.36	0.042
5	7	-0.66	0.531
5	8	0.72	0.491
5	9	0.37	0.714
5	10	-0.68	0.511
5	11	0.74	0.475
5	12	-0.24	0.817
5	13	1.29	0.223
5	14	-0.96	0.375
5	15	-1.95	0.08
6	7	-0.64	0.532
6	8	-0.4	0.695
6	9	2.74	0.01
6	10	-1.74	0.095
6	11	0.31	0.757
6	12	-1.03	0.316
6	13	-2.01	0.056
6	14	-1.42	0.185
6	15	1.01	0.322
7	8	0.53	0.614
7	9	3.07	0.007
7	10	-0.76	0.466
7	11	-0.25	0.809
7	12	-1.96	0.076
7	13	-0.22	0.83
7	14	0.61	0.561
7	15	-0.21	0.834
8	9	4.12	<0.001
8	10	1.55	0.136
8	11	3.91	0.001
8	12	0.82	0.423
8	13	-0.56	0.583
8	14	-3.25	0.008

8	15	-2.25	0.034
9	10	4.38	<0.001
9	11	2.96	0.005
9	12	4.3	<0.001
9	13	2.89	0.007
9	14	3.31	0.004
9	15	3.97	<0.001
10	11	0.19	0.852
10	12	-0.44	0.667
10	13	0.51	0.618
10	14	-1.93	0.083
10	15	1.58	0.127
11	12	1.56	0.133
11	13	-0.3	0.768
11	14	0.33	0.745
11	15	0.84	0.409
12	13	-1.28	0.217
12	14	-1.37	0.199
12	15	0.07	0.944
13	14	1.01	0.331
13	15	0.94	0.357
14	15	1.33	0.206

Supplementary References

1. Spurgin, L. G. *et al.* Gene conversion rapidly generates major histocompatibility complex diversity in recently founded bird populations. *Mol. Ecol.* **20,** 5213–25 (2011).
2. Baillie, L. Genetic population structure of the Trinidadian guppy (*Poecilia reticulata*) across Trrinidad and Tobago. (Dalhousie University, 2012).
3. Paterson, I. G., Crispo, E., Kinnison, M. T., HendrY, A. P. & Bentzen, P. Characterization of tetranucleotide microsatellite markers in guppy (*Poecilia reticulata*). *Mol. Ecol. Notes* **5,** 269–271 (2005).
4. Watanabe, T., Yoshida, M., Nakajima, M. & Taniguchi, N. Isolation and characterization of 43 microsatellite DNA markers for guppy (*Poecilia reticulata*). *Mol. Ecol. Notes* **3,** 487–490 (2003).
5. Shen, X., Yang, G. & Liao, M. Development of 51 genomic microsatellite DNA markers of guppy (*Poecilia reticulata*) and their application in closely related species. *Mol. Ecol. Notes* **7,** 302–306 (2006).
