# Supplementary material for: Evolutionary genetics of immunological supertypes reveals two faces of the Red Queen
Source: Nat Commun. 2017 Nov 3;8:1294. doi: 10.1038/s41467-017-01183-2 (PMC5670221; doi:10.1038/s41467-017-01183-2)
Supplement: Supplementary file 2 — Description of Additional Supplementary Files [file 41467_2017_1183_MOESM2_ESM.pdf]

File Name: Supplementary Data 1
Description: **Supertype designations among identified MHC IIb alleles.**

File Name: Supplementary Data 2
Description: **Location and genotype information for each sample.** For each individual, the specific MHC IIb alleles, which comprise the genotype are given, alongside the supertype group that each allele belongs to. The total number of alleles (*Ai*), and supertypes (*STi*) are also given.

File Name: Supplementary Data 3
Description: **Comparisons of metrics that define each supertype across populations of guppies.**

File Name: Supplementary Data 4
Description: **The frequency of each allele per supertype per population.** The cumulative frequency of a supertypes alleles is also given. Alleles are counter shaded to show those that translate into identical Protein Binding Region amino acids sequences.

File Name: Supplementary Data 5
Description: **Pairwise population estimates of D_{est} based microsatellite allele frequencies.**

File Name: Supplementary Data 6
Description: **Pairwise population estimates of D_{est} based MHC allele frequencies.**

File Name: Supplementary Data 7
Description: **Pairwise population estimates of D_{est} based MHC supertype frequencies**

File Name: Supplementary Data 8
Description: **Microsatellite genotypes**
